# Experimental and Computational Exploration of Chitin, Pectin, and Amylopectin Polymers as Efficient Eco-Friendly Corrosion Inhibitors for Mild Steel in an Acidic Environment. Kinetic, Thermodynamic, and Mechanistic Aspects

**DOI:** 10.3390/polym15040891

**Published:** 2023-02-10

**Authors:** Ahmed Fawzy, Arafat Toghan, Nada Alqarni, Moataz Morad, Magdi E. A. Zaki, Moustafa M. S. Sanad, Abbas I. Alakhras, Ahmed A. Farag

**Affiliations:** 1 Chemistry Department, Faculty of Applied Sciences, Umm Al-Qura University, Makkah 21955, Saudi Arabia; 2 Chemistry Department, Faculty of Science, Assiut University, Assiut 71516, Egypt; 3 Chemistry Department, College of Science, Imam Mohammad Ibn Saud Islamic University (IMSIU), Riyadh 11623, Saudi Arabia; 4 Chemistry Department, Faculty of Science, South Valley University, Qena 83523, Egypt; 5 Department of Chemistry, College of Science and Arts in Balgarn, University of Bisha, Bisha 61922, Saudi Arabia; 6 Central Metallurgical Research & Development Institute, P.O. Box 87, Helwan, Cairo 11421, Egypt; 7 Egyptian Petroleum Research Institute (EPRI), Cairo 11727, Egypt

**Keywords:** mild steel, corrosion, carbohydrate polymers, inhibitors, experimental and theoretical studies

## Abstract

Herein, the inhibition impacts of chitin, pectin, and amylopectin as carbohydrate polymers on the corrosion of mild steel in 0.5 M HCl were researched utilizing various experimental and theoretical tools. The acquired outcomes showed that the inhibition efficiencies (% IEs) of the tested carbohydrate polymers were increased by raising their concentrations and these biopolymers acting as mixed-kind inhibitors with major anodic ones. The acquired % IEs values were reduced with rising temperature. The higher % IEs of the tested polymers were inferred via powerful adsorption of the polymeric molecules on the steel surface and such adsorption obeyed the Langmuir isotherm. The computed thermodynamic and kinetic quantities confirmed the mechanism of physical adsorption. The kinetics and mechanisms of corrosion and its protection by polymeric compounds were illuminated. The results obtained from all the techniques used confirmed that there was good agreement with each other, and that the % of IEs followed the sequence: chitin > amylopectin > pectin.

## 1. Introduction

Corrosion inhibitors are recognized as common operative approaches for keeping the surfaces of metals protected against corrosive media aggressiveness [1,2,3,4,5]. Many kinds of chemical compounds have been assessed and utilized as corrosion inhibitors [6,7,8,9,10,11,12,13,14,15,16]. Carbohydrate polymers, a significant type of natural and biological polymer (biopolymers), are environmentally friendly, biodegradable, biocompatible, cheap, highly abundant, renewable, and can be simply modified to create materials with excellent properties. They show diverse structural characters regarding molecular weights, structures of monosaccharides, etc. These variations determine the functional properties of them. Chitin, poly(*N*-acetylglucosamine), is one of the most supreme, plentiful, natural biopolymers on Earth. It is biodegradable in the natural environment over time [17,18]. *Chitin* has several medicinal and industrial uses such as food processing and biotechnological applications [19]. *Pectin* is an acidic hetero-biopolymer that originates from natural plants. It is employed as a gelling and thickening agent in food, medicines, cosmetics, and in various industrial applications [20]. *Amylopectin* is a much-branched biopolymer that is found in plants [21]. It is also utilized in various domains mostly as a thickening agent, stabilizer, etc.

Carbohydrate polymers have been advertised as a class of molecules that can protect metals from corrosion by acting as good inhibitors over other chemicals due to their inherent stability, the presence of multiple adsorption sites, availability, relatively lower cost, and environmentally friendly nature [22,23,24,25,26,27,28,29,30,31,32,33,34]. Corrosion inhibition by carbohydrate polymers has a unique inhibiting mechanism to counteract corrosion through adsorption on the surfaces of metals via specific centers of adsorption and through forming a shielding layer that protects it from aggressive environments. The specific adsorption centers are related to the cyclic rings located in the biopolymers and the presence of heteroatoms such as nitrogen, oxygen, or sulfur atoms which increase the basicity and electron density in biopolymers, which enhance the strength of the adsorption process. Through these centers, biopolymers construct complexes with different metal ions and on the metal surfaces that engage big surface areas, thus covering the surfaces and shielding them from the aggressiveness of corrosive solutions. Additionally, most biopolymers allow long-time usage due to metallic corrosion inhibition. Furthermore, they are water-soluble compounds without the use of organic solvents. The geometrical configuration and functional groups of these biopolymers are the main factors influencing their corrosion-inhibiting effects [29]. In light of the abovementioned facts, some research groups in recent decades reported that carbohydrate polymers were utilized as proficient corrosion inhibitors in diversity media [22,23,24,25,26,27,28,29,30,31,32,33,34]. Phosphorylated chitin was investigated as a corrosion inhibitor for steel in a neutral medium [22]. Pectin was found to be a hopeful green corrosion inhibitor in various corrosive media depending on its source, molecular weight, and degree of esterification [29,30,31]. Moreover, the protecting impact has been progressed through the addition of proper secondary species such as metal cations [3,8,35,36] and halide ions [14,37] with the tested inhibitor by improving the inhibitor absorption on the surface of metal, called the synergistic effect. Such adsorption was understood in light of the interaction between the inhibitor and these species. 

Frequently, mineral acids are widely utilized in various industrial applications such as acidizing processes, water treating, industrial cleaning, steel pickling, the removal of rust in metal finishing, etc. [38]. In petroleum and gas industries, the usage of acidic media is more customary than neutral or alkaline ones. However, acidic environments are highly aggressive towards metallic surfaces, resulting in metal corrosion [8,9,10,11]. In addition, the presence of certain impurities and/or a high level of aeration in the acidic media accelerates corrosion damage [39]. Because of the ferociousness of these acidic media, metal vessels employed in such activities are mostly exposed to corrosion [8,9,10,11] which is regarded as a dangerous problem confronting economics and care. Therefore, there is a need to mitigate and control the confrontational effects of these media on metal vessels. Instead, mild (SABIC) steel is broadly utilized in various construction applications, infrastructures, and so forth, but it still suffers from corrosion attack, which is regarded as a substantial economic and safety concern. Therefore, the present study aims to explore, for the first time, the performance of three carbohydrate polymers, namely, chitin, pectin, and amylopectin (their structures are illustrated below), as green, cheap, and biocompatible inhibitors in mild-steel corrosion in HCl solutions at a fixed temperature (303 K). Hydrochloric acid is the most important mineral acid used in many industrial applications. For this purpose, several experimental and theoretical tools were used. The thermodynamic and kinetic parameters were computed and are discussed. The kinetics and mechanisms of steel corrosion and its inhibition were also examined and are discussed.

## 2. Experimental Section

### 2.1. Materials

**Figure 1:**
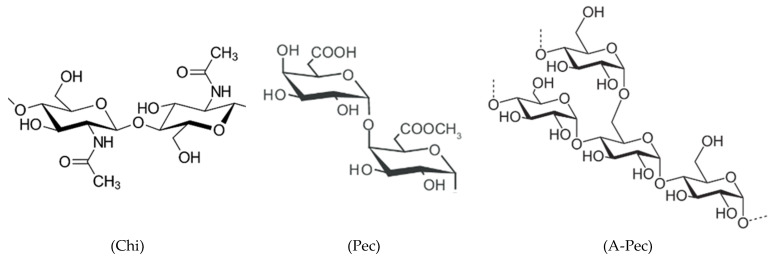
Structures of repeating units of chitin (Chi), pectin (Pec), and amylopectin (A-Pec).

The HCl solution (corrosive medium) was made from Merck 37% HCl. The investigated biopolymers (inhibitors) in this exciting work were three significant carbohydrate polymers (Sigma-Aldrich), namely, chitin, pectin, and amylopectin (Figure 1). Fresh solutions of the investigated inhibitors were made in double-distilled water, which were applied at concentrations of 100, 200, 300, 400, and 500 ppm (mg l^−1^). Most experiments were replicated 3 times in the same conditions to ensure the reproducibility of the results. Corrosion tests were carried out on mild-steel samples (Sabic Company, Saudi Arabia). 

### 2.2. Techniques

Various experimental and theoretical tools were utilized to perform this work. The experimental tools were electrochemical (PDP and EIS), chemical (WL), and spectroscopic (SEM). The theoretical tools were density functional theory (DFT) calculations and molecular dynamic (MD) simulation studies.

PDP and EIS experiments were performed on a thermostated PGSTAT30 potentiostat–galvanostat. The utilized electrochemical cell was a three-electrode cell [2,3]. The working electrode (mild steel) was immersed in an inhibitor-free corrosive environment (HCl) and/or treated with the required inhibitor concentration until a firm potential was reached. In the PDP experiments, the potential of the working electrode was automatically reformed from −200 mV to +200 mV vs. open circuit potential (OCP) at a scan rate of 1.0 mV/s. Using AC signals at OCP, EIS experiments were performed with a frequency range of 100 kHz to 0.1 Hz and an amplitude of 4.0 mV from peak to peak. 

WL was carried out using mild-steel rods with areas of about 14 cm^2^ which were initially prepared before these experiments as reported earlier [2,3]. 

The surfaces of the examined mild-steel examples were imaged prior to and after insertion in the corrosive medium in absence and presence of a certain concentration of the tested carbohydrate polymers. This imaging was performed using a JEOL scanning electron microscope (SEM), model T-200, with a repetition voltage of 10.0 kV. Additionally, the mild-steel surfaces were prepared before imaging as mentioned elsewhere [2,3].

The density functional theory (DFT) supports the experimental results further (using the Gaussian 09 program and the B3LYP/6-31+G (d,p) basis set). The energies of the frontier molecular orbitals, or the highest occupied molecular orbital (*E*_HOMO_) and lowest unoccupied molecular orbital (*E*_LUMO_), as well as the dipole moment, were computed utilizing quantum chemical calculations for the examined carbohydrate polymers (Chi, Pec, and A-Pec). 

The molecular dynamic (MD) simulation studies assess the interaction between the Fe(110) surface and the inhibitor molecules in the simulated corrosive medium using a 5-atom-thick layer unit cell of the Fe(110) surface. These calculations were performed on a slab with a vacuum layer that was 20 Å × 28 Å with a 25 Å. This container holds 200 water molecules and 1 inhibitor molecule. Data from an MD simulation were obtained using the NVT at 298 K with a 1 fs time step and a 0.5 ns simulation period [40,41]. The temperature was changed using the Berendsen thermostat [42]. The COMPASS forcefield, which is extensively used in corrosion studies, was used in the MD simulation.

## 3. Results and Discussion

### 3.1. OCP Measurements

**Figure 2:**
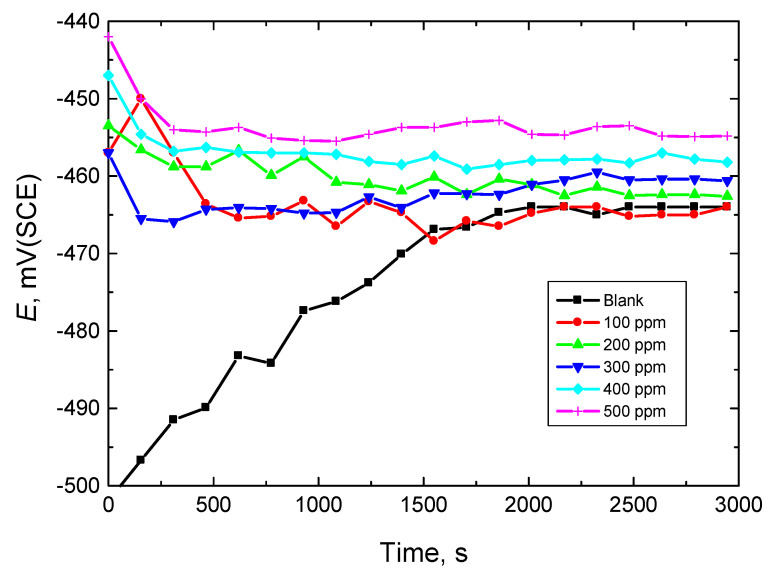
Plots of OCP vs. time for mild steel in 0.5 M HCl solution in absence and presence of numerous amylopectin concentrations at 303 K.

Figure 2 displays the plots of OCP versus time for mild steel in a stagnant 0.5 M HCl solution (corrosive medium) without and with numerous concentrations of amylopectin (as an illustrative example). The figure signifies that the potential of the mild-steel electrode (*E*_OCP_) in the HCl solution moved towards the positive direction up to a time of about 30 min, after which the potential attained a steady state. This behavior indicates the dissolution of the initially air-constructed oxide film resulting in the attack of the metal surface [43]. However, with the addition of amylopectin (A-Pec), *E*_OCP_ began with comparatively greater positive potentials than those in the absence of A-Pec, then moved towards lower positive potentials. The potentials of steady states in the presence of A-Pec were attained rapidly in comparison with the inhibitor-free solution. Additionally, by raising the inhibitor concentration, [A-Pec], the potential of the steady state shifted to a more positive value, suggesting a lower corrosion rate of mild steel and an improvement in the % IE [44]. Moreover, the positive (anodic) shifts in *E*_OCP_ in the presence of A-Pec suggested that such a polymer might behave as an anodic inhibitor. However, because the obtained *E*_OCP_ changes were less than +85 mV, the examined polymers can be considered as mixed-kind inhibitors with an anodic majority [45].

### 3.2. PDP Measurements

**Figure 3:**
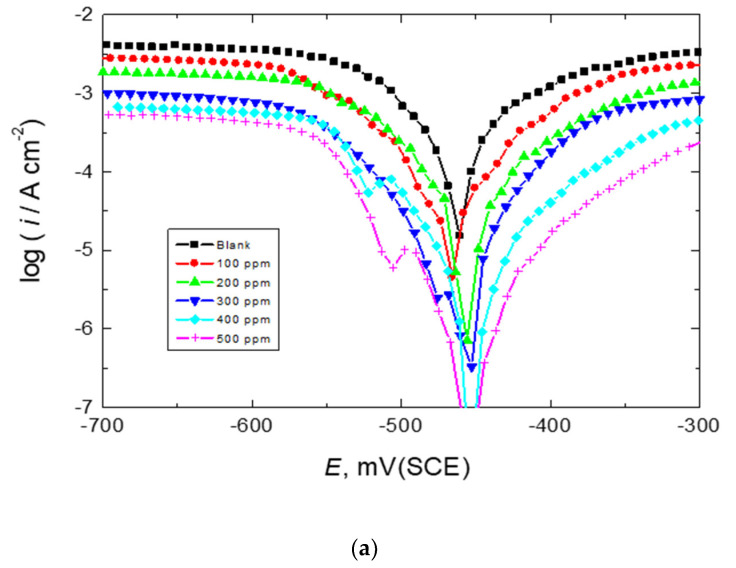

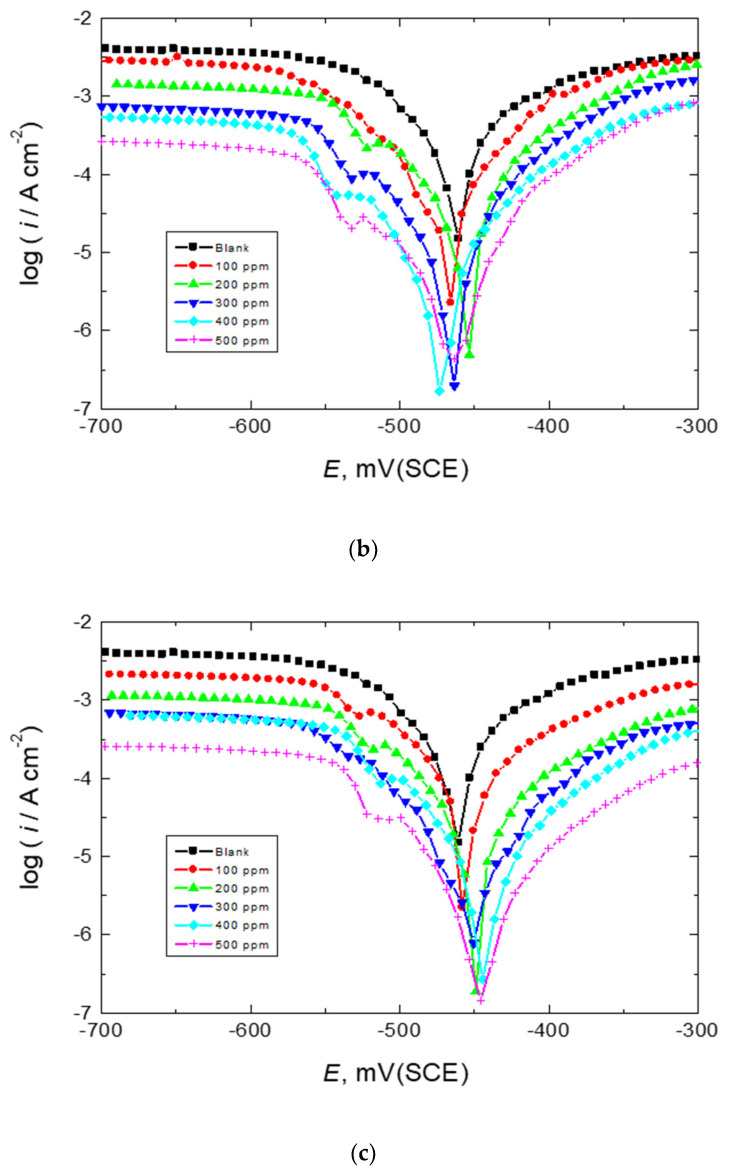
PDP curves (Tafel plots) for mild-steel corrosion in 0.5 M HCl solution at 303 K in absence and presence of the examined carbohydrate polymers: (**a**) Chi, (**b**) Pec, and (**c**) A-Pec.

Figure 3a–c illustrate the PDP curves (Tafel plots) for mild steel in a 0.5 M HCl solution at 303 K, in the absence and presence of 100–500 ppm of the tested carbohydrate polymers, Chi, Pec, and A-Pec, respectively. The values of corrosion potentials (*E*_corr_), anodic and cathodic gradients (*β*_a_, *β*_c_), corrosion current densities (*i*_corr_), polarization resistance (*R*_p_), inhibition efficiencies (% IE), and degrees of surface coverage (θ) of the tested carbohydrate polymers were evaluated and are presented in Table 1. It can be observed that the addition of the studied polymers reduced the *i*_corr_ values, indicating that such polymers are proficient corrosion inhibitors for mild steel in 0.5 M HCl solution. The *E*_corr_ value for steel was somewhat shifted (in most cases) to lower negative values (towards a positive or anodic trend) upon adding the polymers, recommending the mixed-kind inhibition of the tested polymers with anodic seniority (the change in *E*_corr_ was < 85 mV) [45] as discussed in the OCP section. Additionally, both a and c values were found to decrease significantly after the polymers were added, indicating that the polymers reduced anodic metal dissolution and delayed cathodic hydrogen evolution reactions, indicating the polymers’ mixed-kind inhibition. Moreover, the *R*_p_ value was enhanced with increasing the polymers’ concentrations, proving corrosion inhibition. 

The % IE values and θ of the examined polymers (presented in Table 1) were computed from the subsequent equation [46],

(1) $$\%\;\mathrm{IE}=\left[1-\frac{i_{corr}{}_{\left(inh\right)}}{i_{corr}}\right]\;\times \;100=\theta \;\times \;100$$

where *i*_corr_ and *i*_corr(inh)_ point to *i*_corr_ in the absence (blank) and presence of the inhibitor, respectively. The values of % IE were found to augment with raising the polymers’ concentrations and the magnitude of % IEs obeyed the order: chitin > amylopectin > pectin. Overall, it could be concluded that the investigated carbohydrate polymers were proficient mixed-type inhibitors. 

### 3.3. EIS Measurements

**Figure 4:**
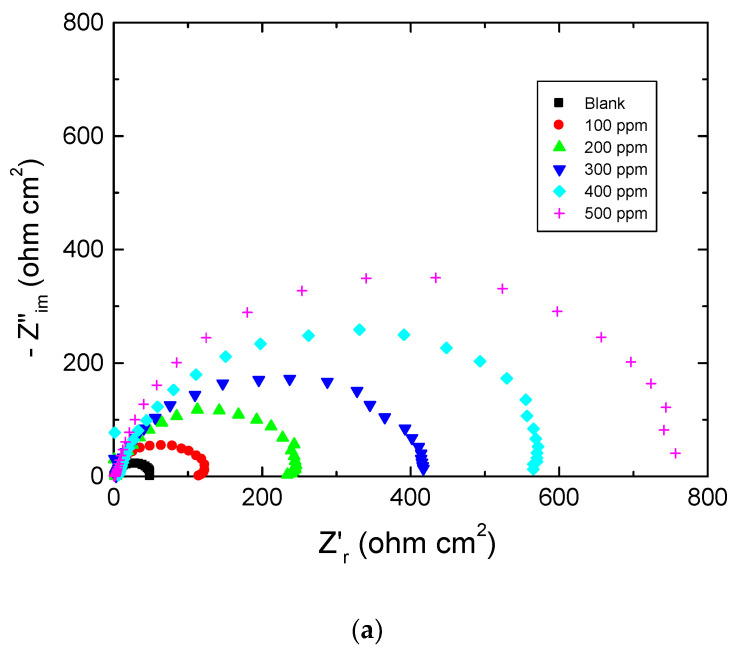

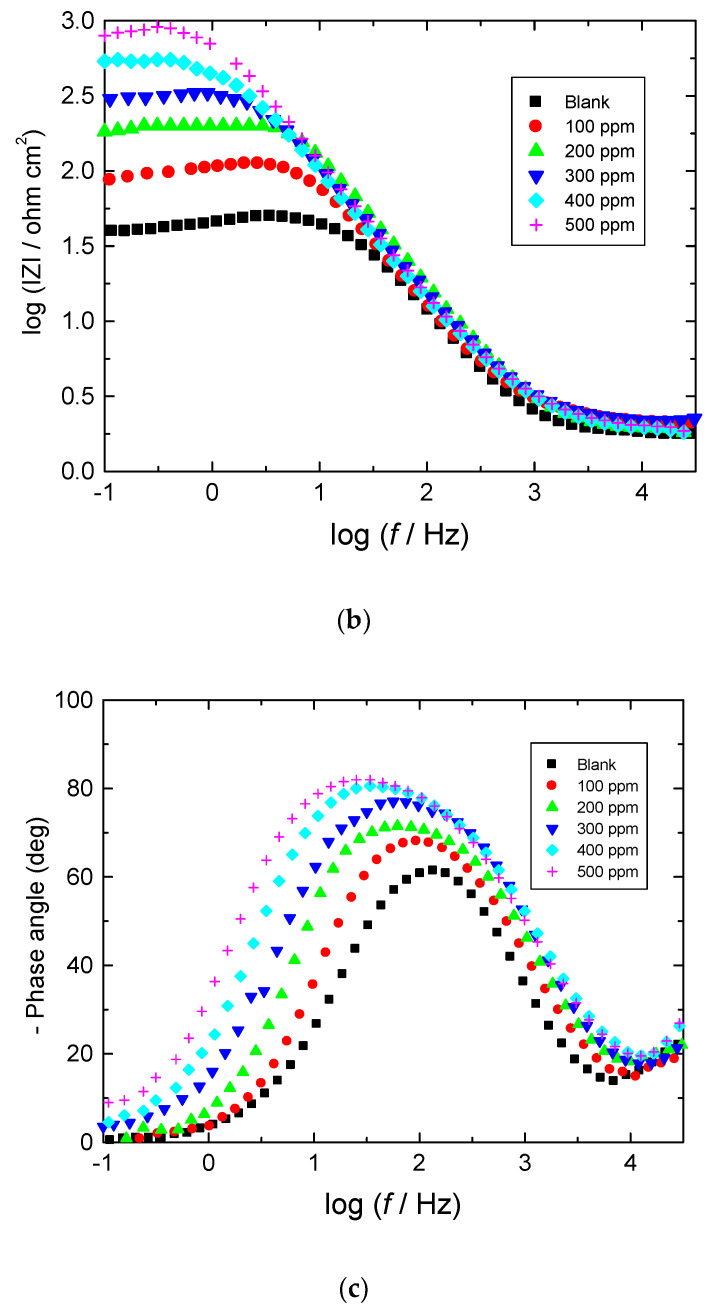
Plots of: (**a**) Nyquist, (**b**) Bode magnitude, and (**c**) Bode phase for mild steel in 0.5 M HCl solution at 303 K in absence and presence of chitin.

**Figure 5:**
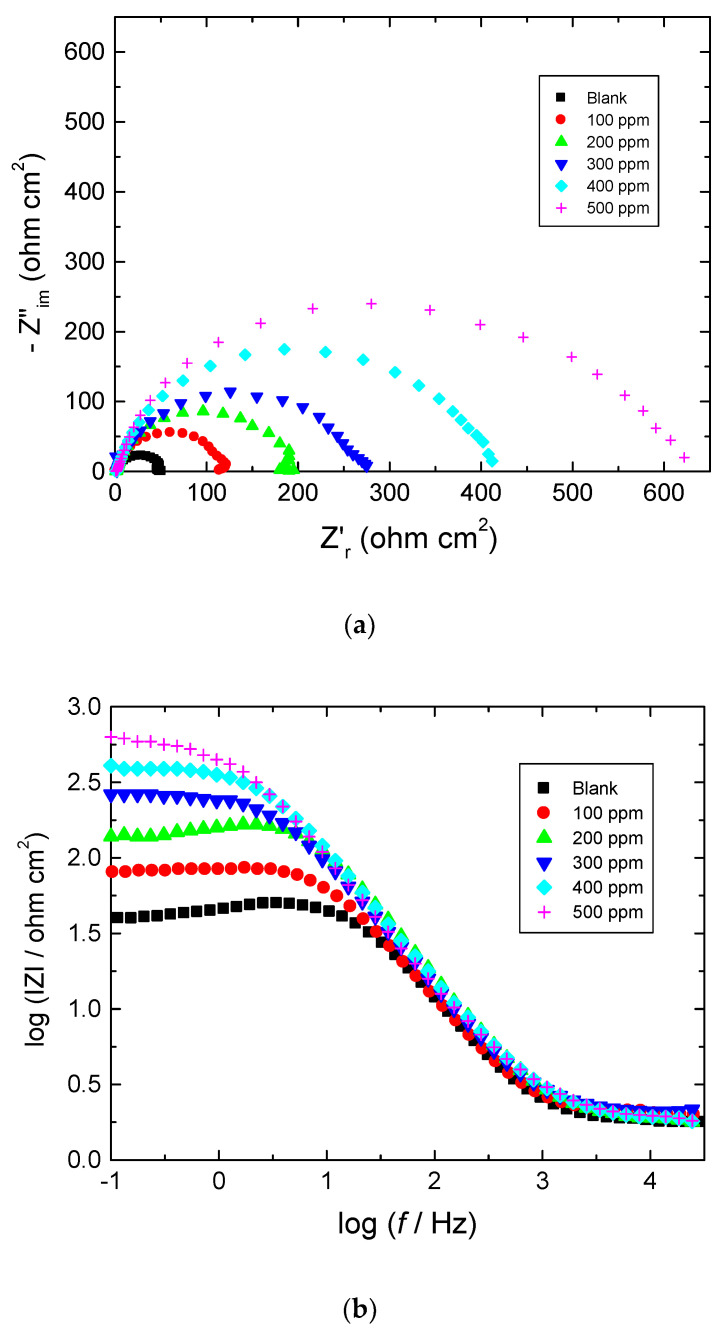

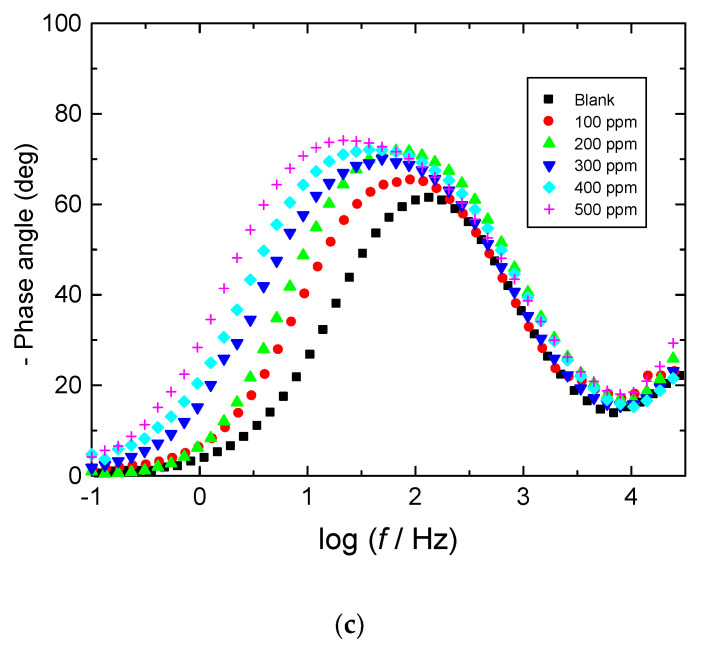
Plots of: (**a**) Nyquist, (**b**) Bode magnitude, and (**c**) Bode phase for mild steel in 0.5 M HCl solution at 303 K in absence and presence of pectin.

**Figure 6:**
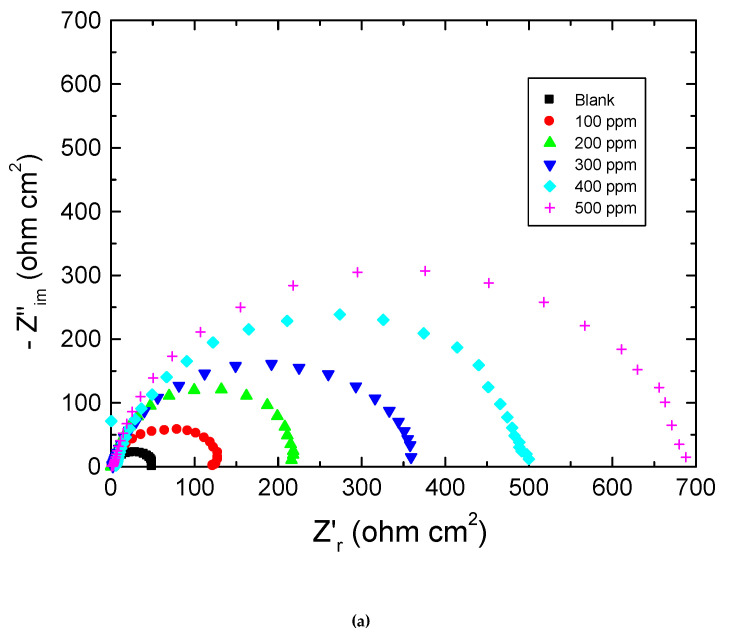

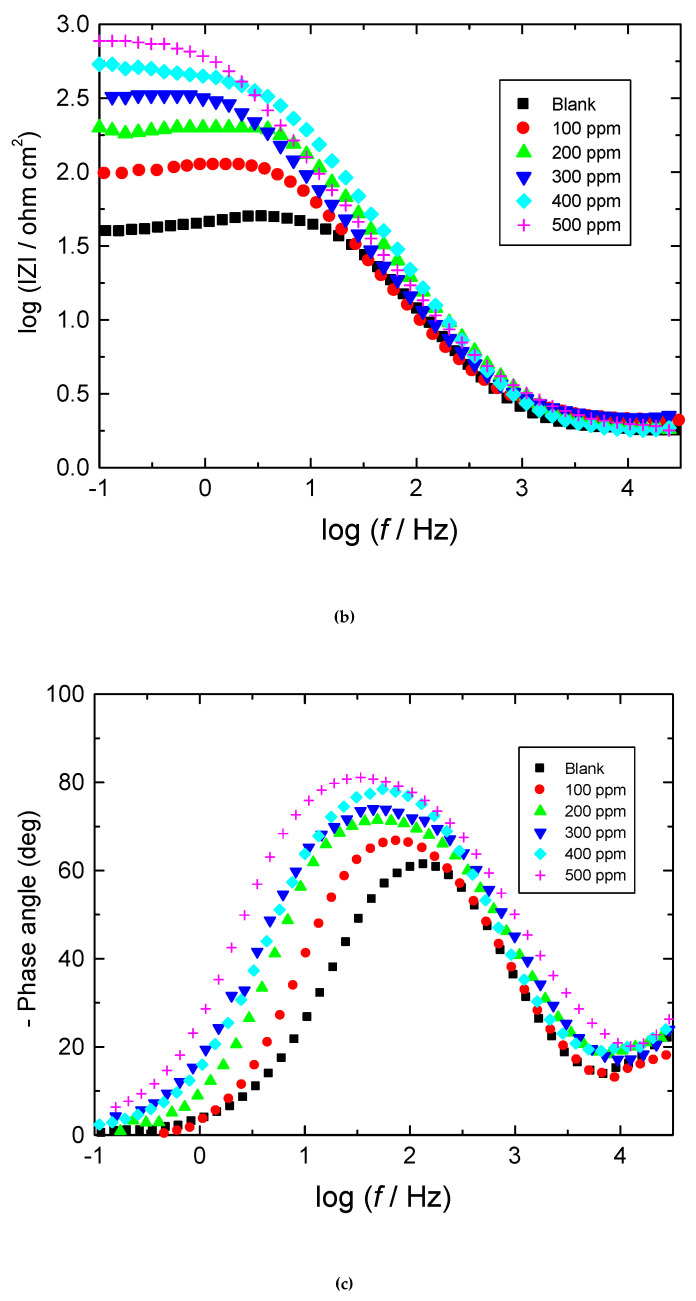
Plots pf: (**a**) Nyquist, (**b**) Bode magnitude, and (**c**) Bode phase for mild steel in 0.5 M HCl solution at 303 K in absence and presence of amylopectin.

**Figure 7:**
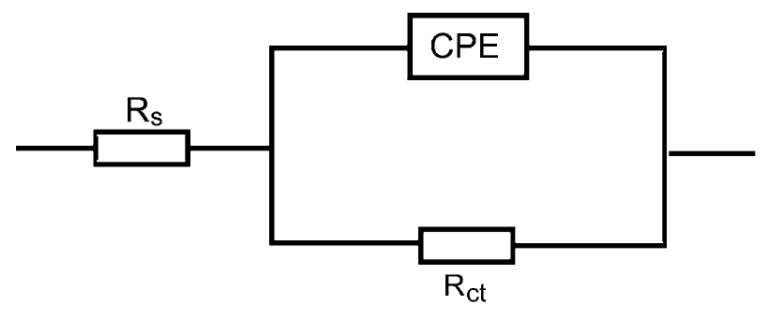
Electrochemical equivalent circuit utilized to fit the EIS output data for mild steel in 0.5 M HCl solution in absence and presence of the examined carbohydrate polymers.

Figure 4, Figure 5 and Figure 6 show: (a) the Nyquist plot and the two forms of Bode plot, (b) the magnitude plot and (c) the phase plot, in the absence and presence of the studied carbohydrate polymers at 303 K. The gained EIS spectra were analyzed via the equivalent circuit presented in Figure 7, similar to that utilized earlier to model the steel/acid interface [47,48]. The components of this circuit were a solution resistance (*R*_s_) and a constant-phase element (CPE), which were presented in the circuit instead of a pure double-layer capacitance to provide a more precise fit and were placed in parallel with charge transfer resistance (*R*_ct_). 

Using CPE points to the heterogeneity of steel surfaces due to surface irregularity, disruptions, impurities, the adsorption of the inhibitor, and the construction of porous adsorption films [49]. 

The EIS parameters, namely, *R*_s_, *R*_ct,_ and CPE, evaluated via EIS spectra are shown in Table 2. The values of % IE were computed from Equation (2) [46] and are also listed in Table 2,

(2) $$\%\;\mathrm{IE}=\left[1-\frac{R_{ct}}{R_{ct(inh)}}\right]\;\times \;100=\theta \;\times \;100$$

The acquired value of *R*_ct_ in the corrosive medium was augmented with increasing polymer concentrations with a reduction in the CPE value, indicating that such polymers reduce the corrosion rate of mild steel. Furthermore, reducing the CPE value implies the adsorption of the polymeric molecules on the metal/solution interface [49] leading to the protection of the metal, thus enhancing the values of the % IEs. 

### 3.4. WL Measurements

#### 3.4.1. Influence of Corrosive Medium

**Figure 8:**
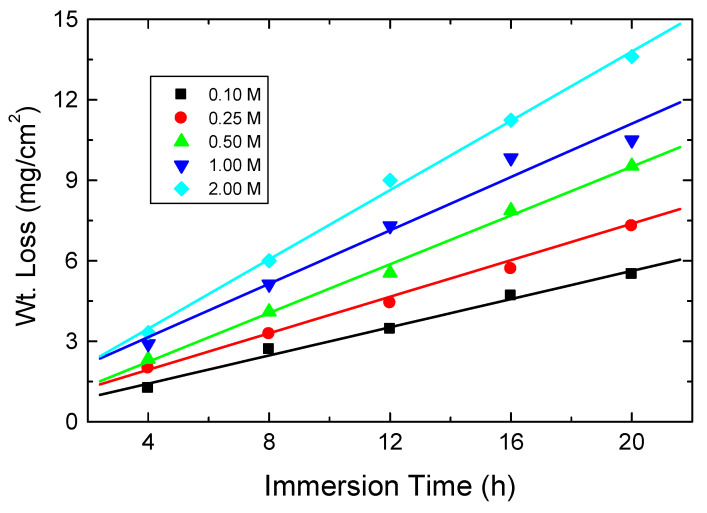
Effect of [HCl] on the CR of mild steel at 303 K.

Figure 8 depicts the weight loss vs. time plots for mild steel at 303 K in various [HCl] concentrations (0.1–2.0 M). The corrosion rates (CR) calculated in mpy are shown in Table 3. The acquired outcomes indicated that the CR of the steel was augmented by increasing [HCl].

#### 3.4.2. Effect of Inhibitors’ Concentrations

**Figure 9:**
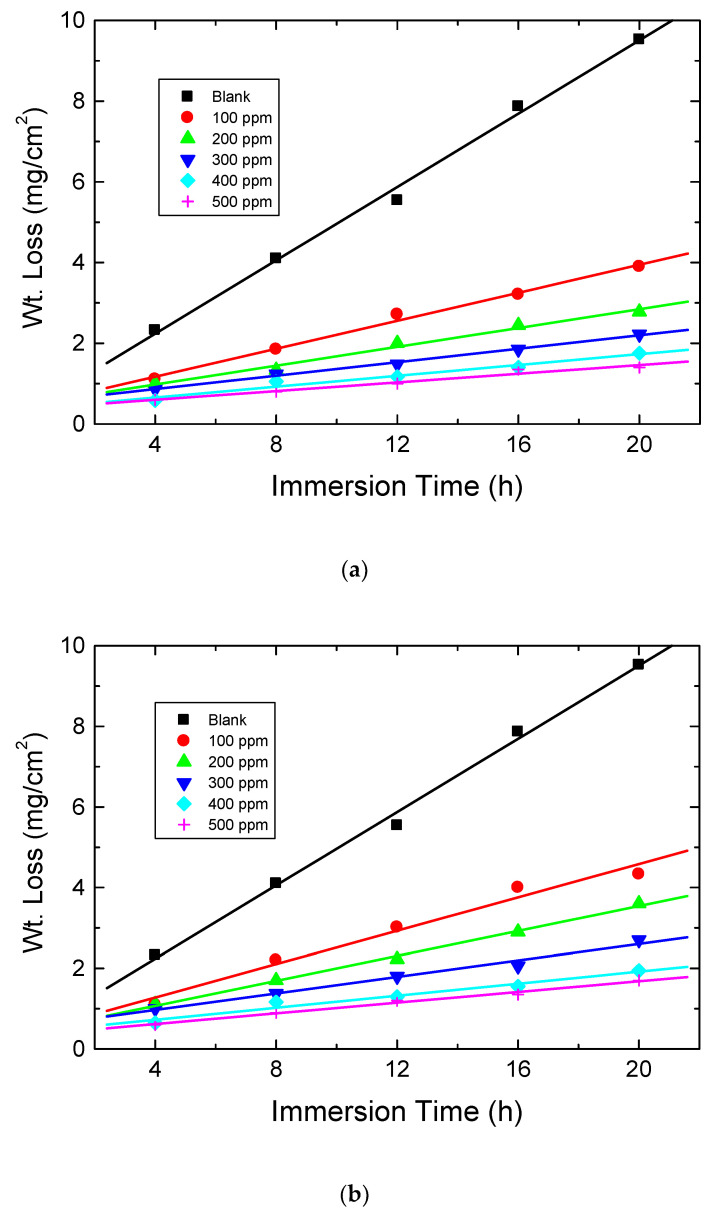

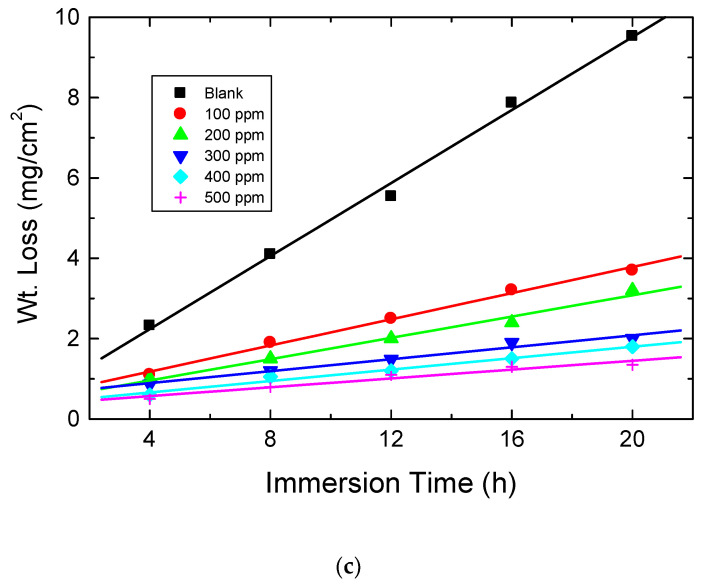
WL vs. time plots for mild steel in 0.5 M HCl solution at 303 K in absence and presence of the examined carbohydrate polymers: (**a**) Chi, (**b**) Pec, and (**c**) A-Pec.

Figure 9 presents the WL runs for mild steel which were carried out in 0.5 M HCl solution (blank) with numerous concentrations (100–500 ppm) of the examined carbohydrate polymers (Chi, Pec, and A-Pec) at 303 K. The CR values for mild steel in the blank and with carbohydrate polymers were calculated and are shown in Table 4. The values of % IEs and θ of these polymers were also computed (Table 4) via Equation (3) [50],

(3) $$\%\;\mathrm{IE}=\left[1-\frac{CR_{inh}}{CR}\right]\;\times \;100=\theta \;\times \;100$$

**Figure 10:**
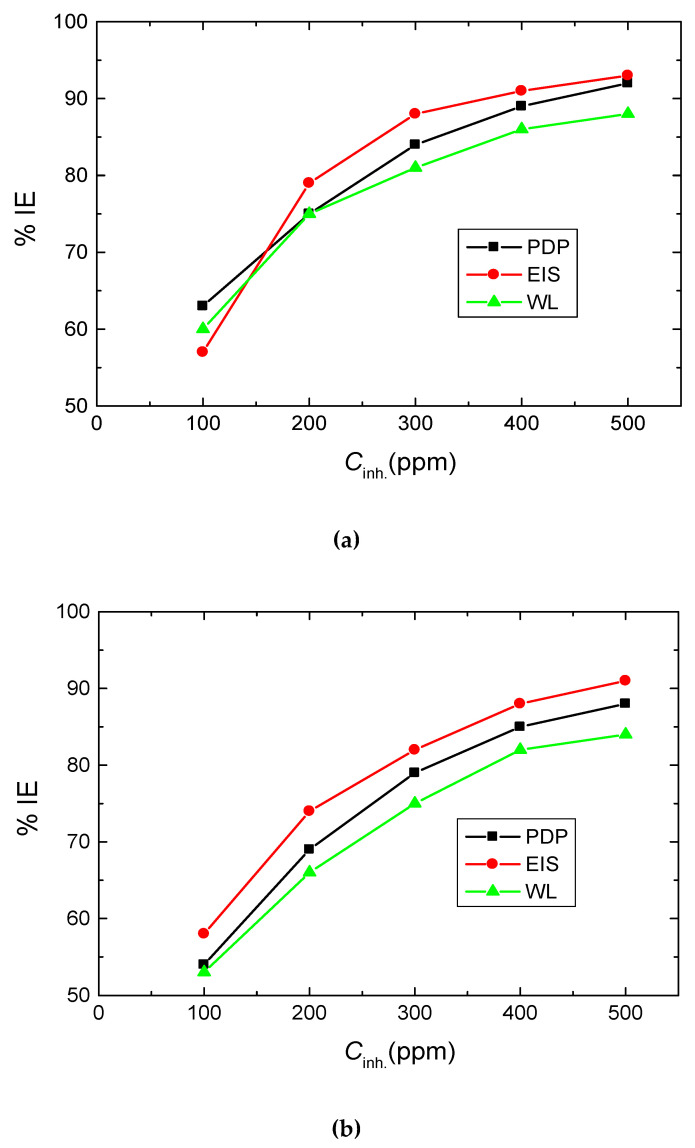

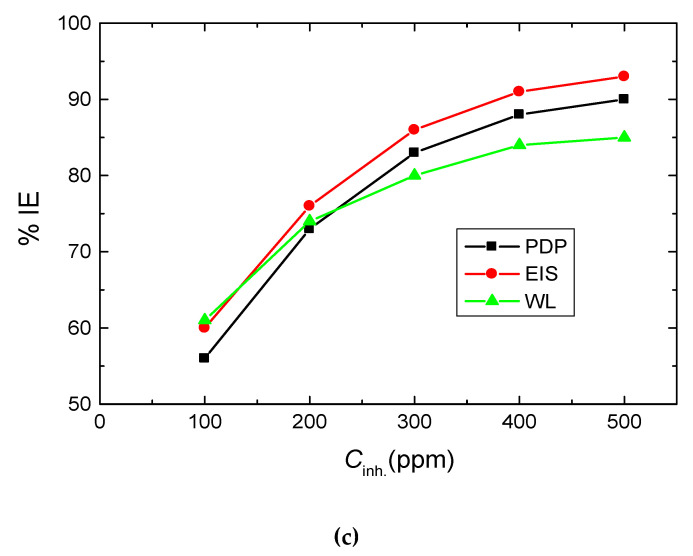
Comparison of different techniques used for evaluation of %IEs of the three polymers tested for inhibition mild-steel corrosion in 0.5 M HCl at 303 K. (**a**) Chi, (**b**) Pec and (**c**) A-Pec.

The obtained results illuminated that adding the tested polymers to the blank reduced the CR of mild steel and, hence, inhibited the rate of steel corrosion. The values of the % IEs of the tested polymers were found to enhance with raising their concentrations. The gained outcomes (Table 4) indicated that, at comparable inhibitor concentrations, the % IE values of the examined carbohydrate polymers were raised in the order: Chi > A-Pec > Pec, in good agreement with those gained from both PDP and EIS tools, proving the rationality of the obtained outcomes as illustrated in Figure 10.

#### 3.4.3. Effect of Time of Immersion on % IEs

**Figure 11:**
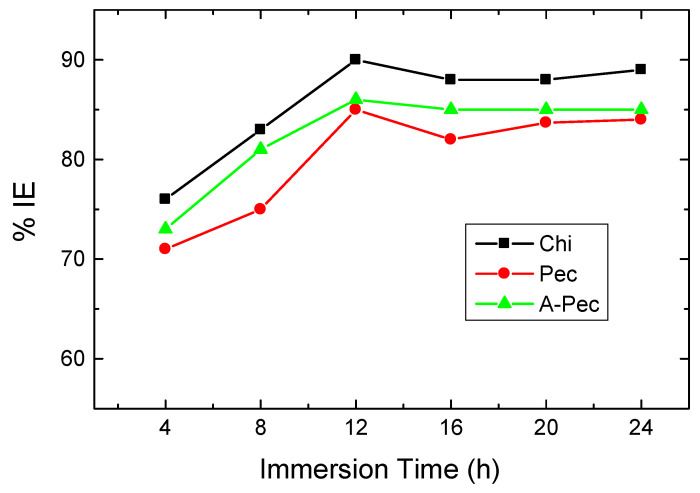
Graphs of the change in %IEs of 500 ppm of the examined carbohydrate polymers with time of immersion for mild steel in 0.5 M HCl at 303 K.

The influence of time of immersion on the % IEs of the tested polymers at a certain concentration (500 ppm as a descriptive case) in 0.5 M HCl solution was explored for 24 h at 303 K as shown in Figure 11. This figure demonstrates that the tested polymeric molecules inhibited mild-steel corrosion for all times of immersion. Initially, the values of % IEs increased continuously with expanding the time of immersion up to around 12 h; afterwards, they reduced slightly for short times and, lastly, they reached approximately the constant values after 16 h. The values of % IEs increasing with the time of immersion at the initial stages can be attributed to the adsorption of multilayers of the polymeric molecules on the steel surface leading to increased % IE values. After around 12 h, some adsorbed polymeric molecules were desorbed from the steel surface, resulting in a reduction in the covered areas with polymeric molecules and, thus, decreasing the % IEs. After 16 h, the constancy of the % IE values with time may be ascribed to the compactness of the adsorbed layers on the steel surface [51].

#### 3.4.4. Effect of Temperature

**Figure 12:**
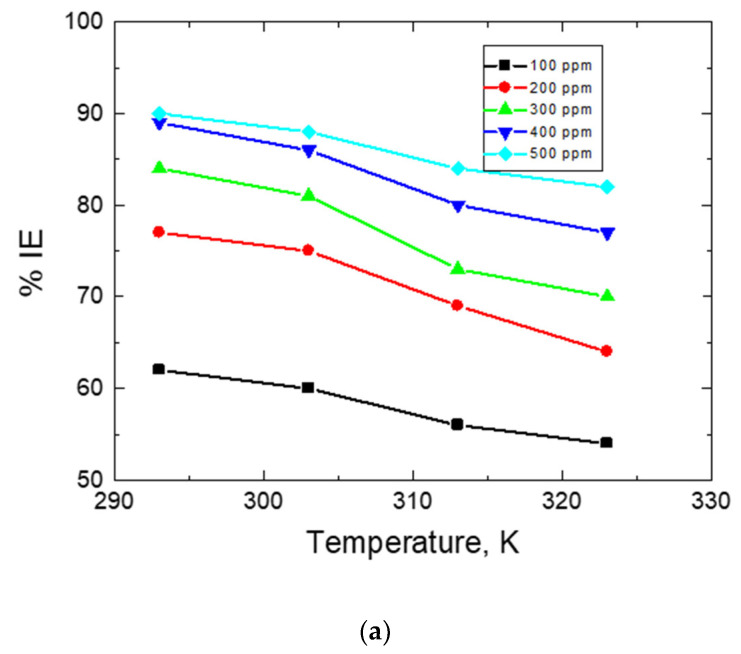

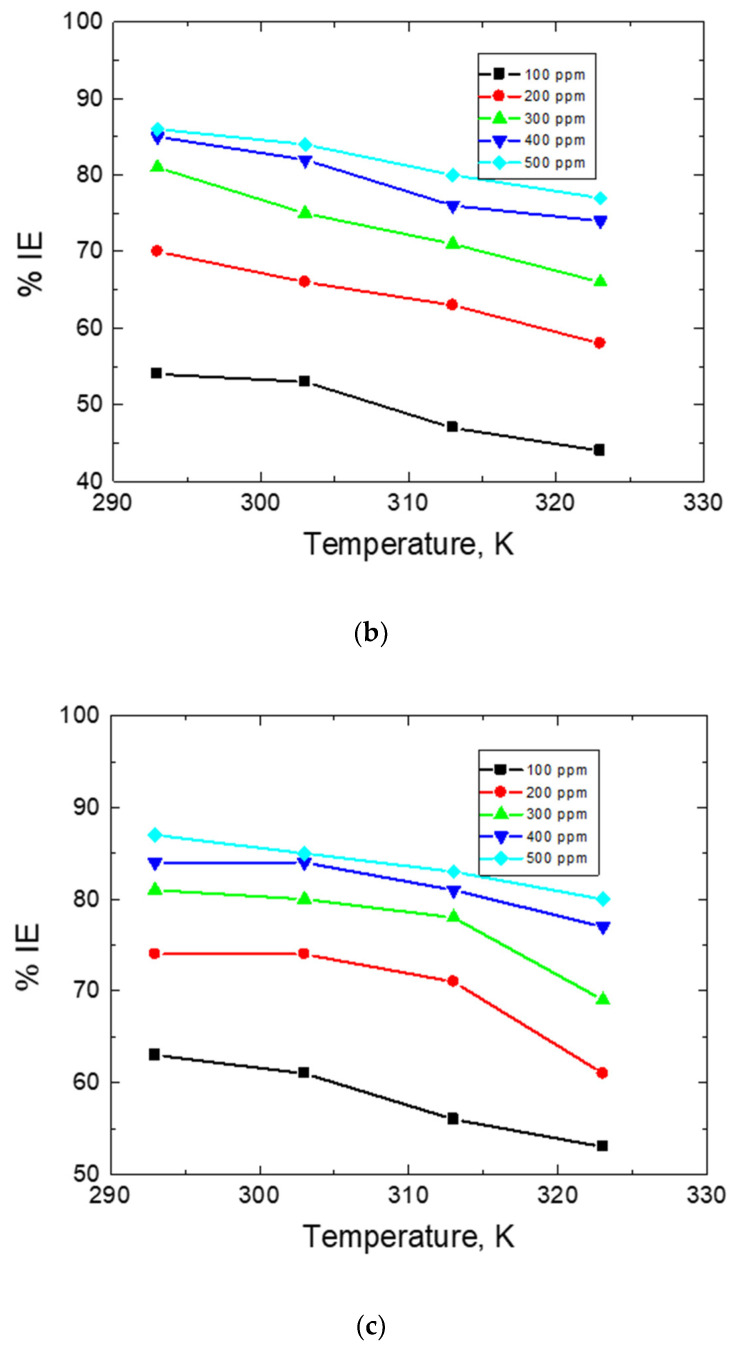
Graphs of the change in %IEs with the temperature in the mild-steel corrosion in 0.5 M HCl comprising various concentrations of the examined carbohydrate polymers. (**a**) Chi, (**b**) Pec, and (**c**) A-Pec.

To evaluate thermodynamic and activation parameters, WL measurements were performed at numerous temperatures. The values of CR of mild steel and both the % IEs and θ values of the tested carbohydrate polymers at different temperatures were evaluated and are shown in Table 4. As the temperature increased, the values of CR increased while the values of IE decreased, as shown in Figure 12. This supports the physical adsorption of the examined polymers [52,53]. 

#### 3.4.5. Adsorption Considerations

**Figure 13:**
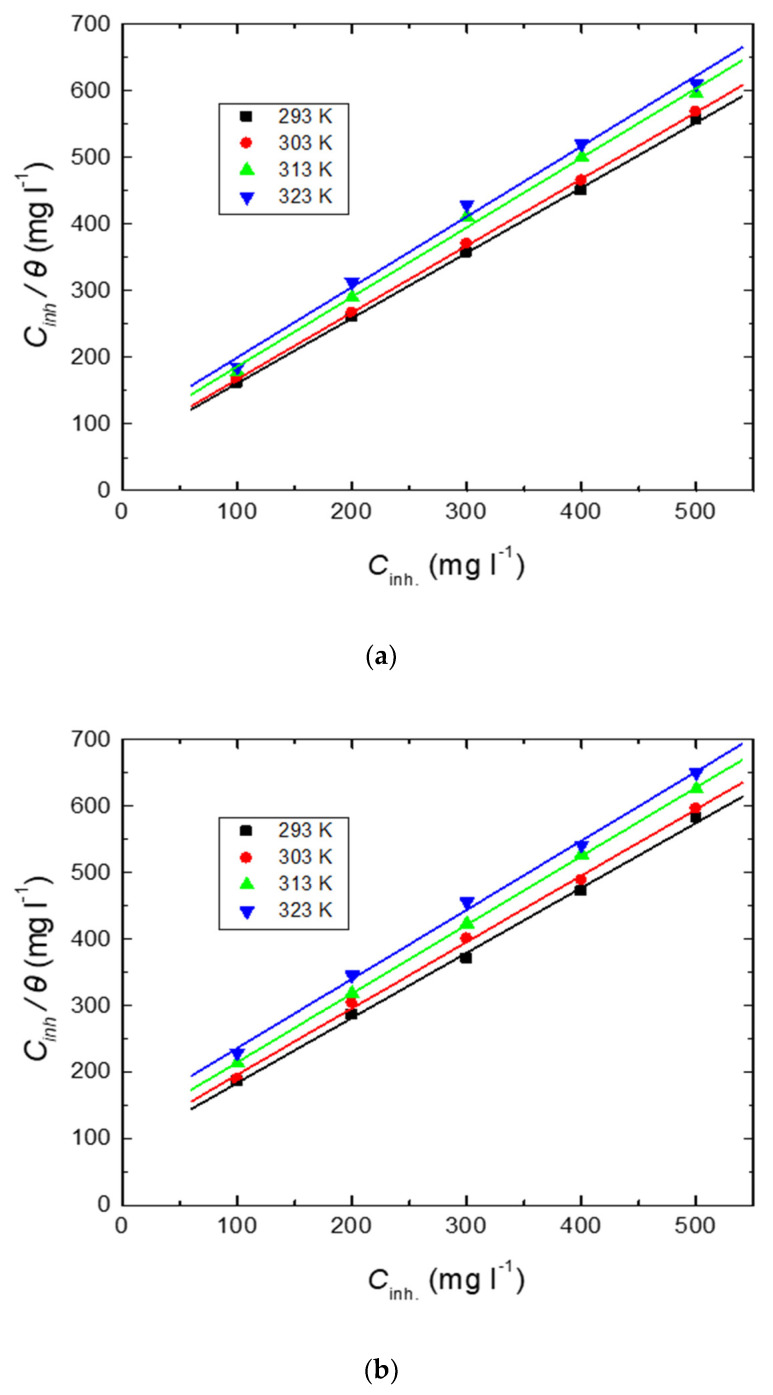

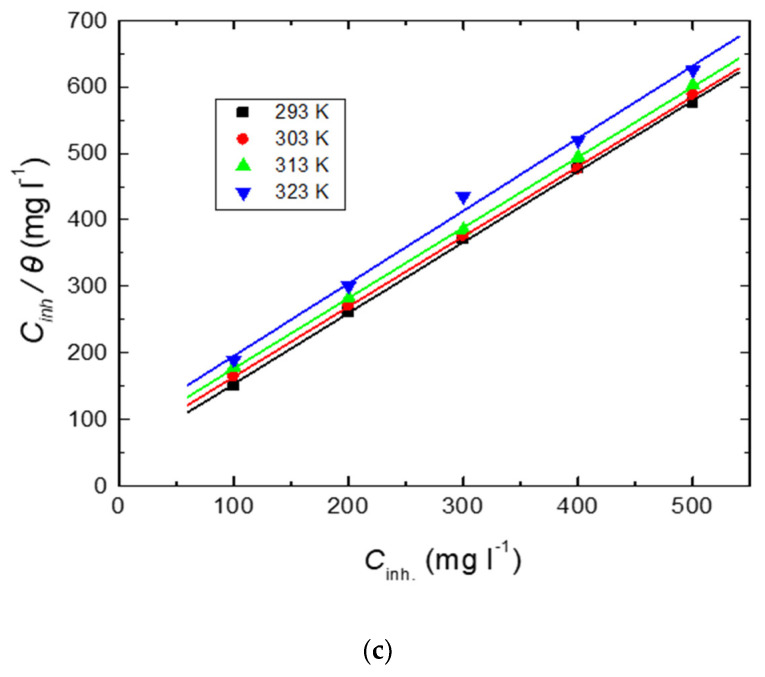
Langmuir adsorption isotherms for the tested carbohydrate polymers: (**a**) Chi, (**b**) Pec, and (**c**) A-Pec adsorbed on mild-steel surface in 0.5 M HCl solution at different temperatures.

In the present investigation, the examined carbohydrate polymers were set to professionally inhibit mild-steel corrosion in a 0.5 M HCl solution up to a % IE of approximately 90%, and such performance was explained by the strong adsorption of the polymeric molecules on the steel surface [54,55,56,57,58]. The illustrative outcomes revealed that the finest depiction of the polymers’ adsorption was the Langmuir isotherm (Figure 13), termed by Equation (4) [59],

(4) $$\frac{C_{inh}^{}}{\theta }=\frac{1}{K_{ads}}+C_{inh}^{}$$

where *K*_ads_ is the adsorption constant. Values of *K*_ads_ were evaluated as the reciprocal of the intercepts of Figure 13 and are presented in Table 5. 

#### 3.4.6. Thermodynamic Parameters

The values of free energy (Δ*G*^o^_ads_), enthalpy (Δ*H*^o^_ads_), and entropy (∆*S*^o^_ads_) of adsorption were evaluated and are presented in Table 5. The Δ*G*^o^_ads_ values were evaluated via Equation (5) [59],

Δ*G*^o^_ads_ = −*RT* ln(55.5 *K*_ads_) (5)

The gained higher negative values of Δ*G*^o^_ads_ designated the spontaneity of adsorption and steadiness of the adsorbed film on the steel surface [60,61]. 

The values of Δ*H*^o^_ads_ were computed via the Van ’t Hoff equation (Equation 6) [62]:

(6) $$\ln\;K_{\mathrm{ads}}=\frac{-\Delta H^{o}{}_{ads}}{RT}+\mathrm{Constant}$$

**Figure 14:**
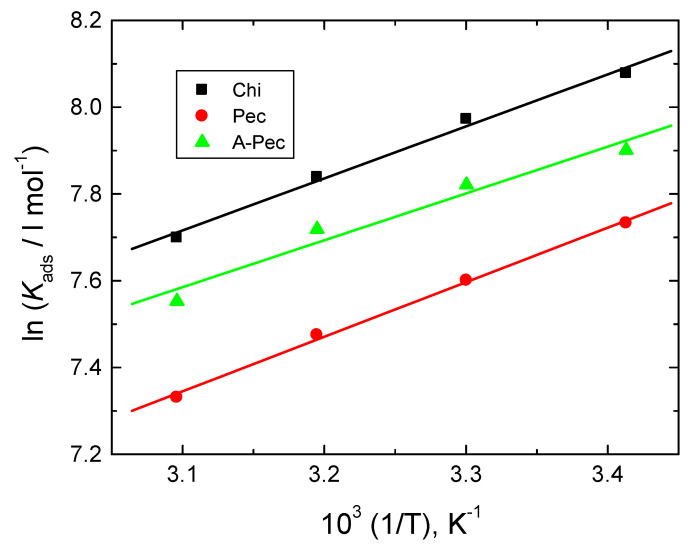
Van ‘t Hoff plots for the examined carbohydrate polymers: (a) Chi, (b) Pec, and (c) A-Pec adsorbed on mild-steel surface in 0.5 M HCl solution.

The ln *K*_ads_ vs. 1/T plots were straight (Figure 14), from which the gained negative values of ∆*H*^o^_ads_ agreed with the exothermic physical adsorption [63].

The ∆*S*^o^_ads_ values were evaluated using the Gibbs–Helmholtz equation, Equation (7)

∆*G*^o^_ads_ = ∆*H*^o^_ads_ −*T*∆*S*^o^_ads_ (7)

The gained positive values of Δ*S*^o^_ads_ specified the bigger disorder of the polymeric molecules in their adsorption on the steel surface [64].

#### 3.4.7. Kinetic Parameters

The values of activation energy (*E*_a_^*^) were evaluated (Table 6) via the Arrhenius equation (Equation 8) [65]:

(8) $$\ln\;\mathrm{CR}=\ln\;A-\frac{E_{a}{}^{*}}{RT}$$

**Figure 15:**
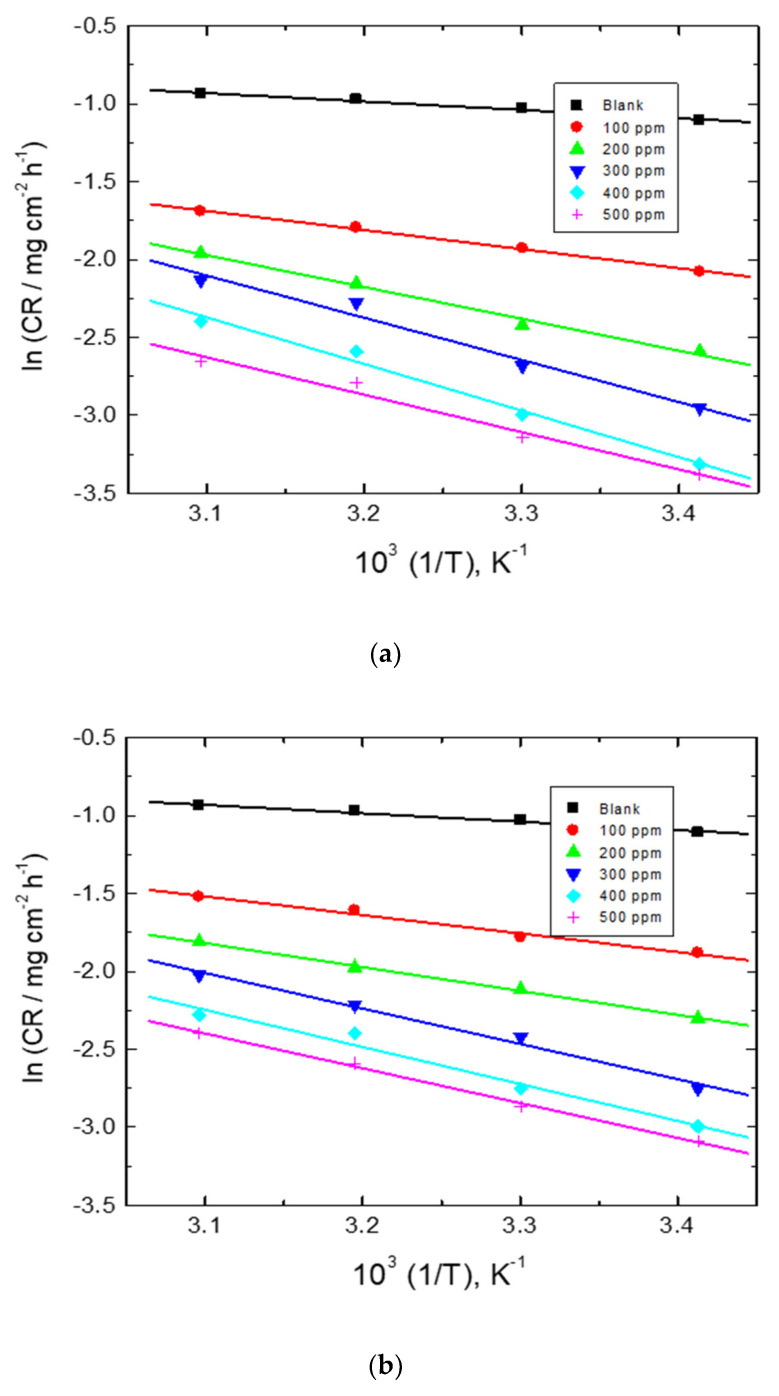

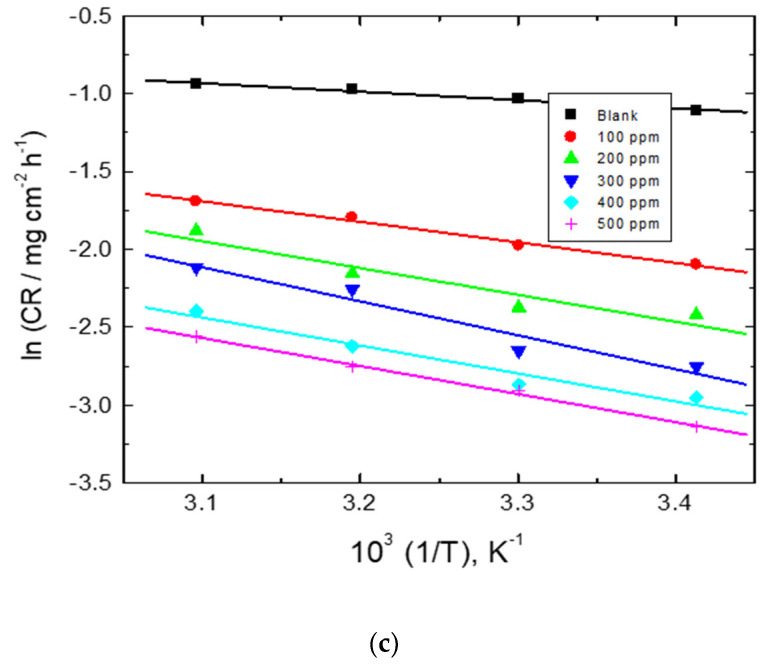
Arrhenius plots for mild-steel corrosion l in 0.5 M HCl solution without and with adding numerous concentrations of the examined carbohydrate polymers: (**a**) Chi, (**b**) Pec, and (**c**) A-Pec.

The Arrhenius plots are illustrated in Figure 15. The gained values of *E*_a_^*^ were within the range of physical adsorption of the polymeric inhibitors [66]. 

The values of both ΔH^*^ and ΔS^*^ were calculated (Table 6) via Equation (9) [67],

(9) $$\ln\left(\frac{CR}{T}\right)=\left(\ln\frac{R}{Nh}+\frac{\Delta S^{*}}{R}\right)-\frac{\Delta H^{*}}{R}\frac{1}{T}$$

**Figure 16:**
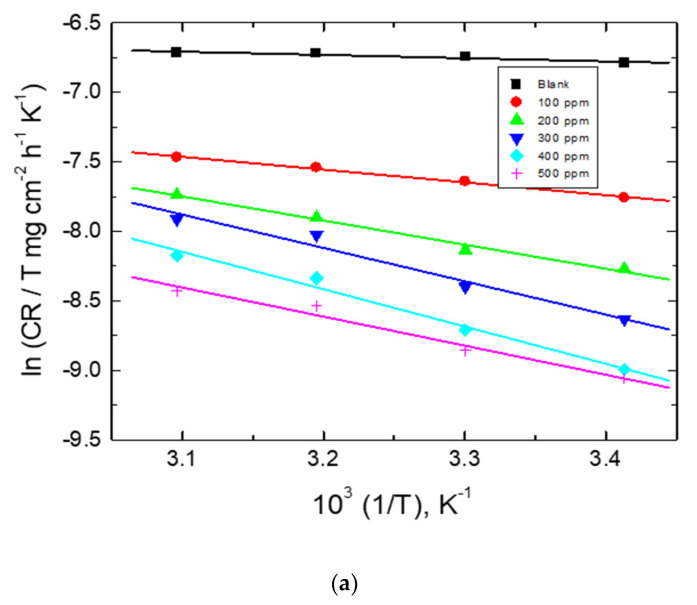

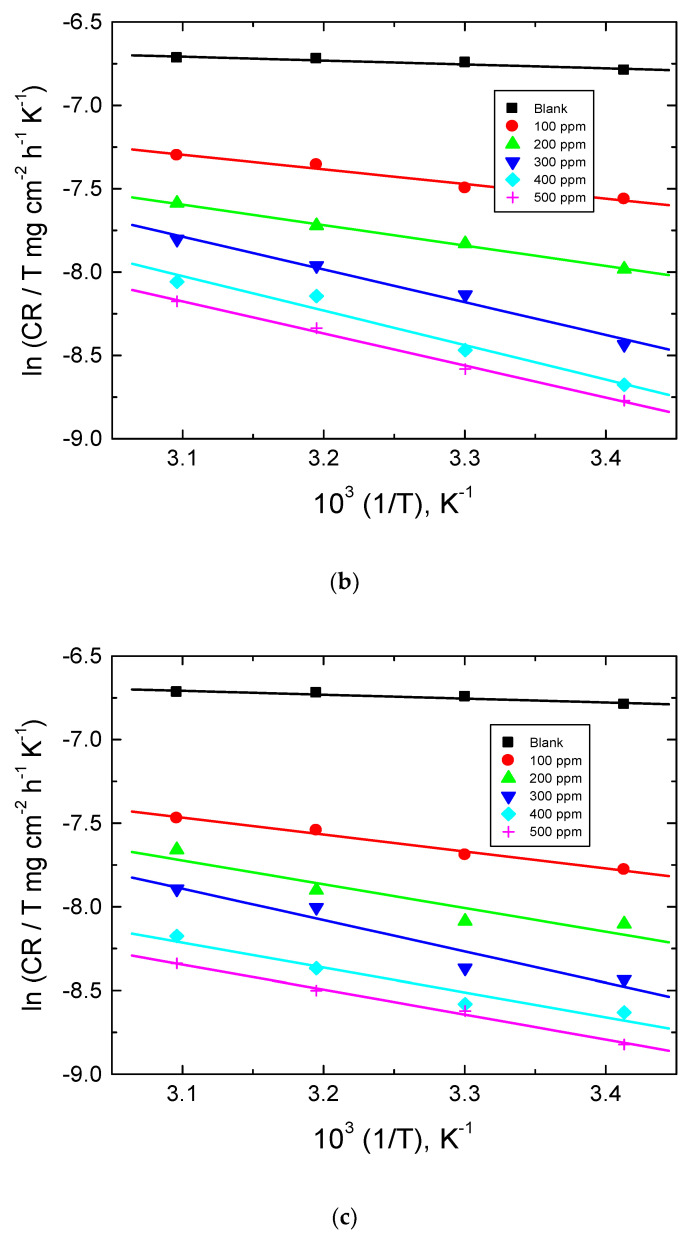
Transition state plots for mild-steel corrosion in 0.5 M HCl solution without and with adding numerous concentrations of the tested carbohydrate polymers: (**a**) Chi, (**b**) Pec, and (**c**) A-Pec.

Also, the transition state plots are shown in Figure 16. The gained positive values of ΔH^*^ refer to the endothermic nature of corrosion, while the negative values of ΔS^*^ describe an association between the polymeric molecules leading to a decrease in the polymeric molecules’ disorder [68].

#### 3.4.8. Kinetics of Corrosion

**Figure 17:**
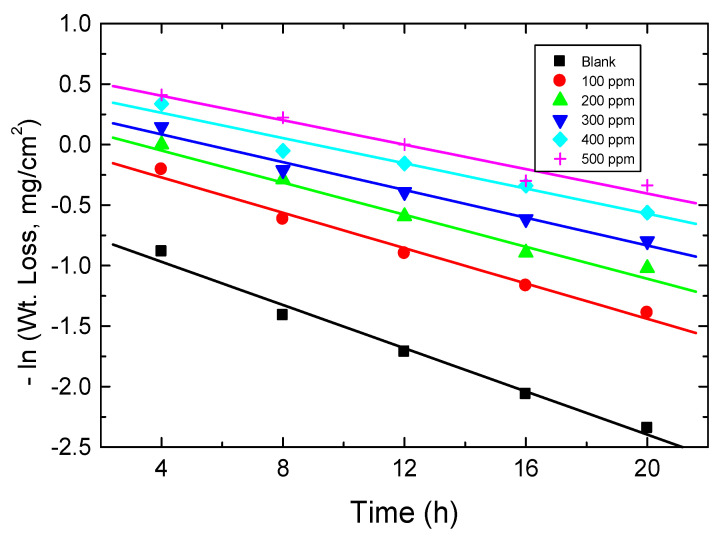
First-order plots for mild-steel corrosion in 0.5 M HCl solution in absence and presence of chitin at 303 K.

The corrosion kinetics of mild steel in a 0.5 M HCl solution were investigated in the absence and presence of various concentrations of the examined carbohydrate polymers. The ln WL vs. time plots (for chitin at 303 K as an illustrative example) were linear (Figure 17), demonstrating that mild-steel corrosion in 0.5 M HCl and its inhibition were negatively first-order reactions. The values of the first-order rate constant, *k*_1_ (in h^−1^), were computed and are shown in Table 7. The values of half-life times (*t*_1/2_, in h) of this process were gained (and are also listed in Table 7) via Equation (10) [69],

(10) $$t_{1/2}=\frac{0.693}{k_{1}}$$

Additionally, the orders (***n***) of corrosion inhibition were calculated using Equation (11) [70],

log CR = log *k* + ***n*** log *C*_inh_ (11)

where *k* is the specific rate constant (mg cm^−2^ h^−1^).

**Figure 18:**
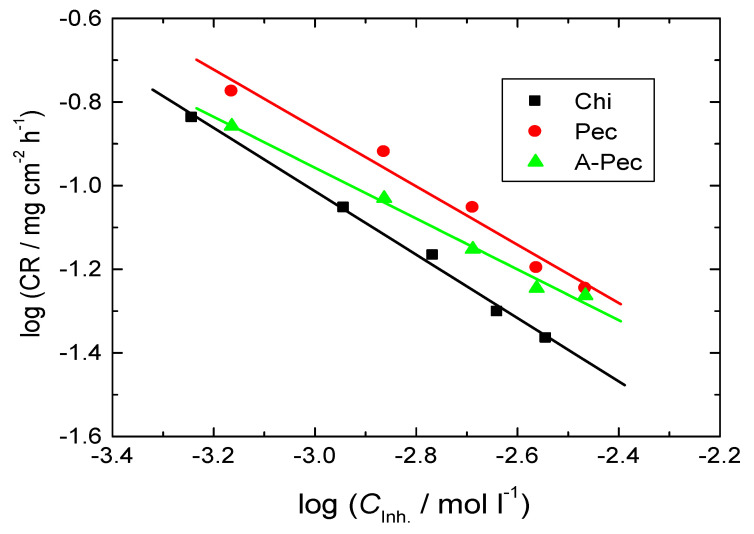
Graphs of log CR vs. log *C*_inh_ for mild-steel corrosion inhibition in 0.5 M HCl solution by the tested carbohydrate polymers at 303 K.

The graphs of log CR vs. log *C*_inh_ for the tested carbohydrate polymers at 303 K were linear as presented in Figure 18. The values of ***n*** were found to be −0.76, −0.69, and −0.61 for Chi, Pec, and A-Pec, respectively (fractional first-order). The negative sign of ***n*** values points to good % IEs [71].

### 3.5. SEM Investigation

**Figure 19:**
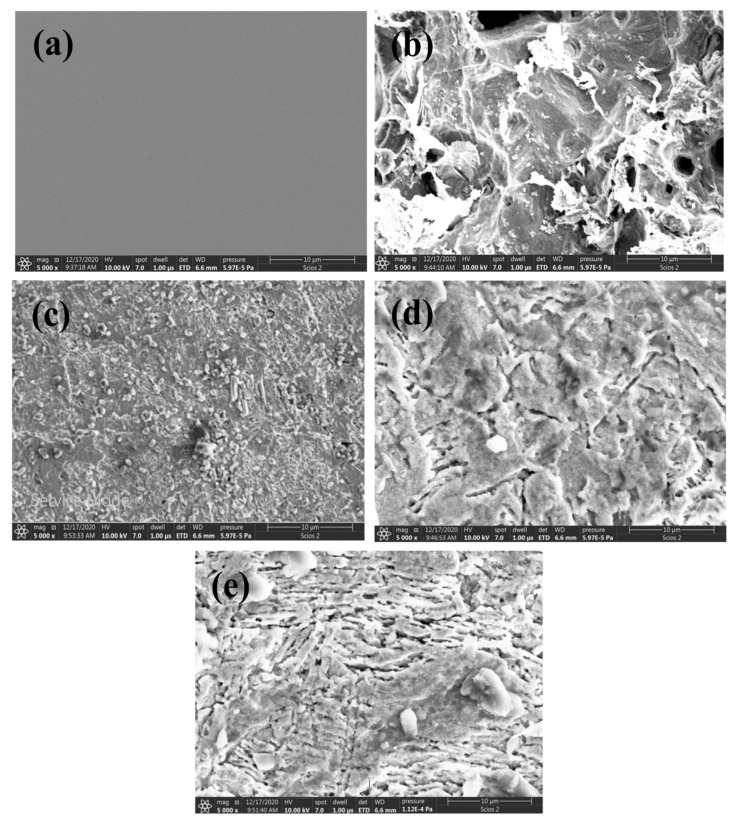
SEM micrographs of mild-steel surfaces; (**a**) prior to immersion, (**b**) after immersion in 0.5 M HCl solution for 24 h, (**c**–**e**) after 24 h immersion in 0.5 M HCl with 300 ppm of the examined carbohydrate polymers, Chi, Pec, and A-Pec, individually.

Figure 19 shows the micrographs of the surfaces of the examined mild-steel specimens prior to and after insertion in the blank (0.5 M HCl) in the absence and presence of 300 ppm of the examined carbohydrate polymers. Figure 19a,b illustrate the surfaces of the mild-steel specimen prior to and after 24 h immersion in the blank, respectively. Figure 19b demonstrates that the surface of the mild steel was highly corroded and various pits were spread on its surface. Figure 19c–e demonstrate the micrographs of the surfaces of the mild-steel specimens after 24 h immersion in the blank with 300 ppm of Chi, Pec, and A-Pec, individually. These micrographs show that the damages shown on the steel surfaces vanished and the surfaces were highly covered with the tested polymers. This is considered proof of the strong adsorption of the polymer molecules on the steel surfaces, thus demonstrating good corrosion inhibition [72].

### 3.6. DFT Study

Every ground state property of an electronic system is exclusively determined by the electron density, according to the Hohenberg Kohn theorem, which forms the foundation of DFT. This hypothesis offers the simplest approach for investigating the molecular structure and behavior of corrosion inhibitors on metal surfaces [73]. The molecules of the investigated carbohydrates polymers contain several oxygen atoms for the Pec and A-Pec moieties as well as several oxygen and nitrogen atoms for the Chi moiety. These heteroatoms might be responsible for the metal surface’s effective adsorption by creating coordinating bonds with the metal ions that prevent corrosion [74]. The polysaccharide polymer molecules can be protonated by the aqueous acidic medium of 0.5 M HCl, which can contribute significantly to the adsorption process.

The Koopman theorem coupled the parameters *E*_HOMO_ and *E*_LUMO_ to ionization potential (*I*) and electron affinity (*A*) values as follows [75]:

(12) $$\mathrm{Ionization}\;\mathrm{potential}\;\left(I\right)=-E_{HOMO}$$

(13) $$\mathrm{Electron}\;\mathrm{affinity}\;\left(A\right)=-E_{LUMO}$$

Other reactivity indices, such as electronegativity (*χ*), electronic chemical potential (*μ*), global hardness (*η*), softness (*σ*), and electron transfer fraction (Δ*N*), were calculated using the formulas:

(14) $$\mathrm{Electronegativity}\;\left(\chi \right)=\frac{I+A}{2}$$

(15) $$\mathrm{Chemical}\;\mathrm{potential}\;\left(\mu \right)=-\chi$$

(16) $$\mathrm{Global}\;\mathrm{hardness}\;\left(\eta \right)=\frac{I-A}{2}$$

(17) $$\mathrm{Softness}\;\left(\sigma \right)=\frac{1}{\eta }$$

(18) $$\mathrm{Electron}\;\mathrm{transfer}\;\mathrm{fraction}\;\left(\Delta N\right)=\frac{\left(\chi_{\mathrm{Fe}}-\chi_{\mathrm{Inh}}\right)}{2\left(\eta_{\mathrm{Fe}}+\eta_{\mathrm{Inh}}\right)}$$

Iron has a global hardness ($\eta_{\mathrm{Fe}}$) of 0 eV and an electronegativity ($\chi_{\mathrm{Fe}}$) of 4.82 eV. Fe is coupled to the Fe (110) surface at 4.82 eV, which has a packed surface and a larger stabilization energy [76]. The value for bulk Fe atoms using Pearson’s electronegativity scale was set to zero [77].

**Figure 20:**
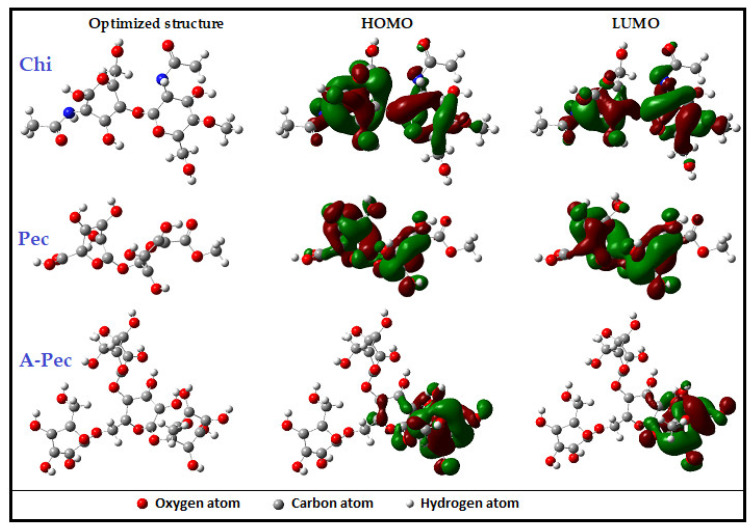
Optimized structure and the frontier molecular orbitals HOMO and LUMO of the tested carbohydrate polymer molecules.

Figure 20 depicts the optimized structure and frontier molecular orbitals, specifically the HOMO and LUMO of Chi, Pec, and A-Pec. The electron density in the HOMO and LUMO orbitals, respectively, appropriately depicts the inhibitor molecule’s electron-donating and electron-accepting sites [78]. The quantum chemical characteristics in Table 8 effectively reveal the corrosion inhibitor’s reactivity as well as how well it adhered to the metal surface. The energy details show how the molecules Chi, Pec, and A-Pec can donate electrons. The higher electron exchange value of Δ*N*, the higher the contact of inhibitors with the metal surface, which improves corrosion inhibition [79]. In conclusion, the Chi inhibitor molecule has a higher propensity to transfer electrons to the metal surface than do the Pec and A-Pec inhibitor molecules. In other words, the molecules of Chi, Pec, and A-Pec bond with the metal surface during the chemisorption process, successfully preventing corrosion [80,81]. Higher dipole moment values are related to improved inhibitor–metal surface interaction through increased polarizability and effective surface area [82]. Compared to the dipole moment of water, the dipole moments of Chi, Pec, and A-Pec are significantly higher (1.88 Debye). As a result, the adsorbed water molecules on the metal surface are successfully replaced by the Chi, Pec, and A-Pec molecules [83].

### 3.7. MD Simulation Study

**Figure 21:**
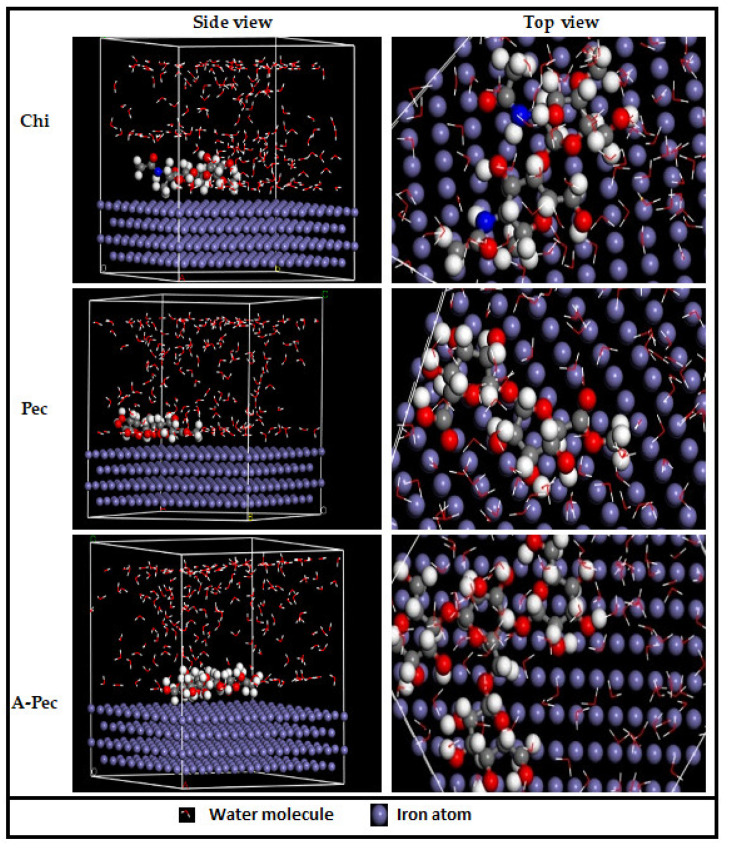
Equilibrium adsorption configurations of the studied carbohydrate polymer molecules on the Fe (110) surface obtained with molecular dynamic simulations.

The MD simulation accurately modelled the interaction between the inhibitor molecules and the metal surface [84]. The Chi, Pec, and A-Pec polymer molecules, as well as the Fe(110) crystal structure, were all adjusted before the MD simulations began. The top and side viewpoints of the interaction site under examination are shown in Figure 21. It can be observed that all of the polymer molecules of carbohydrates were adsorbed on the smooth Fe (110) surface. As a result, the flat location ensured the best contact possible between the heteroatoms and the metallic surface. All inhibitor molecules rejected water molecules from their adsorption sites, implying that as inhibitor concentrations grow, more water molecules will be desorbed from the mild-steel surface [85]. In theory, when the adsorption energy is the lowest during the simulation process, the inhibition performance is at its maximum. The adsorption energies of the Chi, Pec, and A-Pec molecules were determined to be 136387, 136396, and 136515 kcal mol^−1^, respectively, which corresponds to the order of inhibition efficiency reported in the experimental investigations. The negative outcome suggested that the adsorptive system was stable, and spontaneous adsorption may occur in this setting. In general, the theoretical analyses back up the experimental findings.

### 3.8. Mechanism of Corrosion and Corrosion Inhibition

In HCl solutions, the mechanism of corrosion of iron and steel has been suggested by Mulle [86] to proceed according to the following stages:

Anodic reactions which result in the dissolution of iron into ferrous cations as follows,

Fe + Cl^−^ ⇌ (FeCl^−^)_ads_ (19)

(FeCl^−^)_ads_ ⇌ (FeCl)_ads_ + e^−^ (20)

(FeCl)_ads_ → (FeCl^+^)_ads_ + e^−^ (21)

(FeCl^+^)_ads_ → Fe^2+^ + Cl^−^ (22)

Simultaneously, the cathodic reactions occur leading to H_2_ evolution,

Fe + H^+^ ⇌ (FeH^+^)_ads_ (23)

(FeH^+^)_ads_ + e^−^ → (FeCl)_ads_ (24)

(FeH)_ads_ + H^+^ + e^−^ → Fe + H_2_ (25)

Instead, iron and steel can form various oxide phases on their surfaces, which somewhat defend them. The presence of Cl^−^ ions as well as the augmented potential in the positive path, which is applied through the PDP measurements, can dissolve these oxides [33]. When Cl^−^ ions were present in the solution, they strongly attacked the surface of iron as an active anodic potential, leading to the continuous dissolution of iron in the forms of uniform and pitting corrosion [33].

The results obtained from the various tools used in this paper indicated that the inhibitory performance of the investigated carbohydrate polymers on mild-steel corrosion in HCl solutions was determined to be dependent on the chemical structures and concentrations of such polymers. The proposed mechanism of steel corrosion inhibition in the tested medium included strong adsorption of the polymeric molecules on the steel surface due to such compounds containing free electron pairs on the heteroatoms which exist in their chemical structures that can construct coordination bonds with the vacant *d*-orbitals on the iron surface [87]. Furthermore, in the hydrochloric acid solutions, Cl^−^ ions were specifically adsorbed on the steel surface which formed negative charges on the surface. Additionally, in the acidic solutions, the examined polymeric molecules which contained basic groups were suggested to protonate, forming positively charged ones. Therefore, an electrostatic attraction was suggested to occur amongst the positive-charged polymeric molecules and the negative-charged steel surface (physical adsorption), leading to the formation of a strongly adsorbed layer that protected the metal surface [63].

## 4. Conclusions

The inhibitory impacts of chitin, pectin, and amylopectin as carbohydrate polymers on mild-steel corrosion in 0.5 M HCl were explored utilizing several experimental and theoretical techniques. The outcomes of the PDP study showed that the examined polymers were set to be mixed-kind inhibitors with a major anodic one. The high % IEs of the tested polymers were explained via strong polymeric adsorption on the steel surface and such adsorption agreed with the Langmuir isotherm. The computed thermodynamic and kinetic parameters confirmed the mechanism of physical adsorption of the inhibitors. The kinetics and mechanisms of corrosion and its inhibition by the investigated polymers were examined and discussed. The results gained from all employed tools were found to be consistent with each other, which revealed that, under similar experimental circumstances, the inhibition efficiencies of the tested polymers followed the sequence: chitin > amylopectin > pectin.

## Figures and Tables

**Table 1:** Corrosion parameters for mild-steel corrosion in 0.5 M HCl solution in absence and presence of the examined carbohydrate polymers at 303 K.

Inhibitor	Inhibitor Concn. (ppm)	-Ecorr (mV(SCE))	βa (mV/dec.)	-βc (mV/dec.)	icorr (µA/cm^2^)	Rp (ohm cm^2^)	% IE	θ
	0	464	98	103	344	63	--	--
Chi	100	465	76	79	127	132	63	0.63
	200	463	79	88	86	263	75	0.75
	300	461	66	78	55	324	84	0.84
	400	458	76	76	38	486	89	0.89
	500	455	74	77	28	564	92	0.92
Pec	100	469	73	77	158	103	54	0.54
	200	452	76	72	107	172	69	0.69
	300	462	78	64	72	224	79	0.79
	400	472	79	63	50	307	85	0.85
	500	470	77	65	41	388	88	0.88
A-Pec	100	461	96	82	151	127	56	0.56
	200	455	95	79	93	237	73	0.73
	300	452	85	78	58	305	83	0.83
	400	449	93	75	41	456	88	0.88
	500	448	90	76	34	527	90	0.90

**Table 2:** Impedance parameters for mild-steel corrosion in 0.5 M HCl solution in absence and presence of the examined carbohydrate polymers at 303 K.

Inhibitor	Inhibitor Concn. (ppm)	Rs (ohm cm^2^)	Rct (ohm cm^2^)	CPE (µF/cm^2^)	% IE	θ
	0	1.28	51	312	--	--
Chi	100	2.03	118	149	57	0.57
	200	4.12	243	82	79	0.79
	300	4.62	418	54	88	0.88
	400	7.34	595	44	91	0.91
	500	3.20	756	39	93	0.93
Pec	100	1.92	122	130	58	0.58
	200	2.74	198	81	74	0.74
	300	3.91	276	61	82	0.82
	400	6.34	412	57	88	0.88
	500	6.81	595	51	91	0.91
A-Pec	100	2.27	128	124	60	0.60
	200	4.62	213	93	76	0.76
	300	3.70	355	56	86	0.86
	400	9.33	543	45	91	0.91
	500	6.05	696	41	93	0.93

**Table 3:** Values of CR for mild steel in diverse [HCl] solutions at 303 K.

[HCl], M	0.10	0.25	0.50	1.00	2.00
CR (mpy)	118	142	177	199	247

**Table 4:** Values of CR of mild steel, %IE, and θ of the tested carbohydrate polymers, Chi, Pec, and A-Pec, in 0.5 M HCl solution at diverse temperatures.

Inhibitor	Inhibitor Concn. (ppm)	Temperature (K)											
		293			303			313			323		
		CR	% IE	θ	CR	% IE	θ	CR	% IE	θ	CR	% IE	θ
	0	145	--	--	157	--	--	166	--	--	172	--	--
Chi	100	55	62	0.62	64	60	0.60	73	56	0.56	81	54	0.54
	200	33	77	0.77	39	75	0.75	51	69	0.69	62	64	0.64
	300	23	84	0.84	30	81	0.81	45	73	0.73	52	70	0.70
	400	16	89	0.89	22	86	0.86	33	80	0.80	40	77	0.77
	500	15	90	0.90	19	88	0.88	27	84	0.84	31	82	0.82
Pec	100	67	54	0.54	74	53	0.53	88	47	0.47	96	44	0.44
	200	44	70	0.70	53	66	0.66	61	63	0.63	72	58	0.58
	300	28	81	0.81	39	75	0.75	48	71	0.71	58	66	0.66
	400	22	85	0.85	28	82	0.82	40	76	0.76	45	74	0.74
	500	20	86	0.86	25	84	0.84	33	80	0.80	40	77	0.77
A-Pec	100	54	63	0.63	61	61	0.61	73	56	0.56	81	53	0.53
	200	40	74	0.74	41	74	0.74	51	71	0.71	67	61	0.61
	300	28	81	0.81	31	80	0.80	46	78	0.78	53	69	0.69
	400	23	84	0.84	25	84	0.84	32	81	0.81	40	77	0.77
	500	19	87	0.87	24	85	0.85	28	83	0.83	34	80	0.80

**Table 5:** Thermodynamic parameters and *K*_ads_ for mild-steel corrosion in 0.5 M HCl solution in the absence and presence of the examined carbohydrate polymers at diverse temperatures.

Inhibitor	Temp. (K)	10^−3^ *K*_ads_ l mol^−1^	∆*G*^o^_ads_ kJ mol^−1^	∆*H*^o^_ads_ kJ mol^−1^	∆*S*^o^_ads_ J mol^−1^ K^−1^
**Chi**	293	3.22	−29.06	−9.97	133.21
	303	2.91	−29.78		131.19
	313	2.54	−30.42		129.04
	323	2.20	−31.02		126.90
**Pec**	293	2.28	−28.40	−10.47	132.66
	303	1.99	−29.04		130.40
	313	1.76	−29.67		128.23
	323	1.53	−30.23		126.01
**A-Pec**	293	2.70	−29.03	−8.98	129.73
	303	2.38	−29.65		127.49
	313	2.08	−30.27		125.40
	323	1.91	−31.09		124.06

**Table 6:** Activation parameters for mild-steel corrosion in 0.5 M HCl solution in the absence and presence of the examined carbohydrate polymers.

Inhibitor	Inhibitors Concn. (mg l^−1^)	*E*_a_^*^ kJ mol^−1^	∆H^*^ kJ mol^−1^	∆S^*^ J mol^−1^ K^−1^
	**0**	**4.49**	**1.93**	**−49.88**
**Chi**	**100**	10.23	7.65	−38.24
	**200**	16.96	6.69	−19.95
	**300**	22.45	19.95	−3.66
	**400**	24.94	22.28	−1.41
	**500**	19.87	17.37	−15.96
**Pec**	**100**	9.89	7.32	−38.24
	**200**	12.72	10.16	−31.59
	**300**	18.87	16.29	−14.13
	**400**	19.70	17.13	−13.30
	**500**	18.54	15.96	−18.29
**A-Pec**	**100**	10.97	8.40	−35.75
	**200**	14.38	11.81	−27.43
	**300**	18.12	15.54	−16.62
	**400**	14.96	12.31	−29.93
	**500**	14.84	12.39	−30.76

**Table 7:** Values of *k*_1_ and *t*_1/2_ for mild-steel corrosion in 0.5 M HCl solution in the absence and presence of chitin at 303 K.

Inhibitors Concn. (mg l^−1^)	10^3^ *k*_1_, h^−1^	*t*_1/2_, h
0 (Blank)	89	7.79
100	73	9.49
200	66	10.50
300	57	12.16
400	51	13.59
500	49	14.14

**Table 8:** Quantum chemical parameters for the tested carbohydrate polymers.

Parameters	Chi	Pec	A-Pec
EHOMO (ev)	−0.140	−0.164	−0.153
ELUMO (ev)	−0.134	−0.135	−0.108
Energy gap (ev)	0.006	0.029	0.046
Ionization potential (I)	0.140	0.164	0.153
Electron affinity (A)	0.134	0.135	0.108
Electronegativity (χ)	0.137	0.149	0.130
Global hardness (η)	0.003	0.014	0.023
Global softness (σ)	335.008	69.686	43.802
ΔN	784.451	162.736	102.705
Dipole moment (D)	14.283	6.297	8.088

## Data Availability

Data presented in this study are available on request from the corresponding author.

## References

[1] Toghan A., Fawzy A., Al Bahir A., Alqarni N., Sanad M.M.S., Khairy M., Alakhras A.I., Farag A.A. (2022). Computational Foretelling and Experimental Implementation of the Performance of Polyacrylic Acid and Polyacrylamide Polymers as Eco-Friendly Corrosion Inhibitors for Copper in Nitric Acid. Polymers.

[2] Fawzy A., Abdallah M., Zaafarany I., Ahmed S., Althagafi I. (2018). Thermodynamic, kinetic and mechanistic approach to the corrosion inhibition of carbon steel by new synthesized amino acids-based surfactants as green inhibitors in neutral and alkaline aqueous media. J. Mol. Liq..

[3] Alqarni N. (2022). Investigation of Expired Ticarcillin and Carbenicillin Drugs for Inhibition of Aluminum Corrosion in Hydrochloric Acid Solution. Int. J. Electrochem. Sci..

[4] El-Lateef H.M.A., Shaaban S., Khalaf M.M., Toghan A., Shalabi K. (2021). Synthesis, experimental, and computational studies of water soluble anthranilic organoselenium compounds as safe corrosion inhibitors for J55 pipeline steel in acidic oilfield formation water. Colloids Surf. A Physicochem. Eng. Asp..

[5] Fawzy A., Abdallah M., Alfakeer M., Ali H.M. (2019). Corrosion inhibition of Sabic iron in different media using synthesized sodium N-dodecyl arginine surfactant. Int. J. Electrochem. Sci..

[6] Farag A.A., Mohamed E.A., Toghan A. (2022). The new trends in corrosion control using superhydrophobic surfaces: A review. Corros. Rev..

[7] El-Lateef H.M.A., El-Beltagi H.S., Mohamed M.E.M., Kandeel M., Bakir E., Toghan A., Shalabi K., Tantawy A.H., Khalaf M.M. (2022). Novel Natural Surfactant-Based Fatty Acids and Their Corrosion-Inhibitive Characteristics for Carbon Steel-Induced Sweet Corrosion: Detailed Practical and Computational Explorations. Front. Mater..

[8] Toghan A., Fawzy A., Alqarni N., Abdelkader A., Alakhras A.I. (2021). Inhibition Effects of Citrulline and Glutamine for Mild Steel Corrosion in Sulfuric Acid Environment: Thermodynamic and Kinetic Aspects. Int. J. Electrochem. Sci..

[9] Toghan A. (2022). Electrochemical and Theoretical Examination of Some Imine Compounds as Corrosion Inhibitors for Carbon Steel in Oil Wells Formation Water. Int. J. Electrochem. Sci..

[10] Hazazi O.A., Fawzy A., Awad M.I. (2015). Sulfachloropyridazine as an eco-friendly inhibitor for corrosion of mild steel in H_2_SO_4_ solution. Chem. Sci. Rev. Lett..

[11] Heikal M., Ali A., Ibrahim B., Toghan A. (2020). Electrochemical and physico-mechanical characterizations of fly ash-composite cement. Constr. Build. Mater..

[12] Bawazeer T.M., El Ghamry H.A., Farghaly T.A., Fawzy A. (2020). Novel 1,3,4 thiadiazolethiosemi-carbazones derivatives and their divalent cobalt complexes: Synthesis, characterization and their efficiencies for acidic corrosion inhibition of carbon steel. J. Inorg. Organomet. Polym. Mater..

[13] Takroni K.M., El-Ghamry H.A., Fawzy A. (2019). Evaluation of the Catalytic Activities of Some Synthesized Divalent and Trivalent Metal Complexes and Their Inhibition Efficiencies for the Corrosion of Mild Steel in Sulfuric Acid Medium. J. Inorg. Organomet. Polym. Mater..

[14] Hazazi O.A., Fawzy A., Awad M.I. (2014). Synergistic effect of halides on the corrosion inhibition of mild steel in H_2_SO_4_ by a triazole derivative: Kinetics and thermodynamic studies. Int. J. Electrochem. Sci..

[15] Abdallah M., Al Jahdaly B.A., Salem M.M., Fawzy A., Abdel Fattah A.A. (2017). Pitting corrosion of nickel alloys and stainless steel in chloride solutions and its inhibition using some inorganic compounds. J. Mater. Environ. Sci..

[16] Abdallah M., Salem M.M., Zaafarany I.A., Fawzy A., Fattah A.A.A. (2017). Corrosion Performance of Stainless Steel and Nickel Alloys in Aqueous Sodium Hydroxide as Revealed from Cyclic Voltammetry and Potentiodynamic Anodic Polarization. Orient. J. Chem..

[17] Ravi Kumar M.N.V. (2000). A review of chitin and chitosan applications. React. Funct. Polym..

[18] Farag A.A., Badr E.A. (2020). Non-ionic surfactant loaded on gel capsules to protect downhole tubes from produced water in acidizing oil wells. Corros. Rev..

[19] Merzendorfer H., Cohen E. (2019). Chitin/Chitosan: Versatile Ecological, Industrial, and Biomedical Applications. Extracellular Sugar-Based Biopolymers Matrices.

[20] Zhao Z.Y., Liang L., Fan X., Yu Z., Hotchkiss A., Wilk B.J., Eliaz I. (2008). The role of modified citrus pectin as an effective chelator of lead in children hospitalized with toxic lead levels. Altern. Ther. Health Med..

[21] Green M.M., Blankenhorn G., Hart H. (1975). Which starch fraction is water-soluble, amylose or amylopectin?. J. Chem. Educ..

[22] Toghan A., Gouda M., Shalabi K., El-Lateef H. (2021). Preparation, Characterization, and Evaluation of Macrocrystalline and Nanocrystalline Cellulose as Potential Corrosion Inhibitors for SS316 Alloy during Acid Pickling Process: Experimental and Computational Methods. Polymers.

[23] Al-Gamal A.G., Farag A.A., Elnaggar E.M., Kabel K.I. (2018). Comparative impact of doping nano-conducting polymer with carbon and carbon oxide composites in alkyd binder as anti-corrosive coatings. Compos. Interfaces.

[24] Al-Sabagh A.M., Abdou M.I., Migahed M.A., Abd-Elwanees S., Fadl A.M., Deibac A. (2018). Investigations using potentiodynamic polarization measurements, cure durability, ultra violet immovability and abrasion resistance of polyamine cured ilmenite epoxy coating for oil and gas storage steel tanks in petroleum sector. Egypt. J. Pet..

[25] Farag A.A., Ismail A.S., Migahed M.A. (2019). Squid By-product Gelatin Polymer as an Eco-friendly Corrosion Inhibitor for Carbon Steel in 0.5 M H2SO4 Solution: Experimental, Theoretical, and Monte Carlo Simulation Studies. J. Bio-Tribo-Corros..

[26] Mohamed H.A., Farag A.A., Badran B.M. (2008). Corrosion inhibitiob of mild steel using emulsified thiazole adduct in Different binder systems. Eurasian Chem. J..

[27] Shaban S.M., a Badr E., Shenashen M., Farag A. (2021). Fabrication and characterization of encapsulated Gemini cationic surfactant as anticorrosion material for carbon steel protection in down-hole pipelines. Environ. Technol. Innov..

[28] Farag A. (2018). Oil-in-water emulsion of a heterocyclic adduct as a novel inhibitor of API X52 steel corrosion in acidic solution. Corros. Rev..

[29] Abdallah M., Fawzy A., Hawsawi H. (2020). Estimation of water-soluble polymeric materials (Poloxamer and Pectin) as corrosion inhibitors for carbon steel in acidic medium. Int. J. Electrochem. Sci..

[30] Fares M.M., Maayta A., Al-Qudah M.M. (2012). Pectin as promising green corrosion inhibitor of aluminum in hydrochloric acid solution. Corros. Sci..

[31] Grassino A.N., Halambek J., Djaković S., Brnčić S.R., Dent M., Grabarić Z. (2016). Utilization of tomato peel waste from canning factory as a potential source for pectin production and application as tin corrosion inhibitor. Food Hydrocoll..

[32] Abdallah M., Fawzy A., Hawsawi H. (2020). Maltodextrin and chitosan polymers as inhibitors for the corrosion of carbon steel in 1.0 M hydrochloric acid. Int. J. Electrochem. Sci..

[33] Fawzy A., Abdallah M., Alfakeer M., Altass H.M., Althagafi I.I., El Ossaily Y.A. (2021). Performance of unprecedented synthesized biosurfactants as green inhibitors for the corrosion of mild steel-37-2 in neutral solutions: A mechanistic approach. Green Chem. Lett. Rev..

[34] Abdallah M., Fawzy A., Alfakeer M., Altass H.M. (2021). Expired azithromycin and roxithromycin drugs as environmentally friendly inhibitors for mild steel corrosion in H_2_SO_4_ solutions. Green Chem. Lett. Rev..

[35] Li X., Deng S., Fu H., Mu G. (2009). Synergistic inhibition effect of rare earth cerium(IV) ion and 3,4-dihydroxybenzaldehye on the corrosion of cold rolled steel in H_2_SO_4_ solution. Corros. Sci..

[36] Fawzy A., Farghaly T.A., Al Bahir A.A., Hameed A.M., Alharbi A., El-Ossaily Y.A. (2020). Investigation of three synthesized propane bis-oxoindoline derivatives as inhibitors for the corrosion of mild steel in sulfuric acid solutions. J. Mol. Struct..

[37] Li X., Tang L., Li L., Mu G., Liu G. (2006). Synergistic inhibition between o-phenanthroline and chloride ion for steel corrosion in sulphuric acid. Corros. Sci..

[38] Williams D.A., Holifield P.K., Looney J.R., McDougall L.A. (1993). Inhibited Acid System for Acidizing Wells. U.S. Patent.

[39] Liu Y., Zhang B., Zhang Y., Ma L., Yang P. (2016). Electrochemical polarization study on crude oil pipeline corrosion by the produced water with high salinity. Eng. Fail. Anal..

[40] Hsissou R., Dagdag O., Abbout S., Benhiba F., Berradi M., El Bouchti M., Berisha A., Hajjaji N., Elharfi A. (2019). Novel derivative epoxy resin TGETET as a corrosion inhibition of E24 carbon steel in 1.0 M HCl solution. Experimental and computational (DFT and MD simulations) methods. J. Mol. Liq..

[41] Farag A.A., Mohamed E.A., Sayed G.H., Anwer K.E. (2021). Experimental/computational assessments of API steel in 6 M H_2_SO_4_ medium containing novel pyridine derivatives as corrosion inhibitors. J. Mol. Liq..

[42] Dagdag O., Hsissou R., El Harfi A., Berisha A., Safi Z., Verma C., Ebenso E., Touhami M.E., El Gouri M. (2020). Fabrication of polymer based epoxy resin as effective anti-corrosive coating for steel: Computational modeling reinforced experimental studies. Surf. Interfaces.

[43] Evans U.R. (1960). The Corrosion of Metals.

[44] Farag A.A., Toghan A., Mostafa M.S., Lan C., Ge G. (2022). Environmental Remediation through Catalytic Inhibition of Steel Corrosion by Schiff’s Bases: Electrochemical and Biological Aspects. Catalysts.

[45] Amin M.A., Khaled K.F. (2010). Monitoring corrosion and corrosion control of iron in HCl by non-ionic surfactants of the TRITON-X series – Part I. Tafel polarisation, ICP-AES and EFM studies. Corros. Sci..

[46] Toghan A., Dardeer H.M., Gadow H.S., Elabbasy H.M. (2021). New promising halogenated cyclic imides derivatives as Potential Corrosion Inhibitors for Carbon Steel in Acidic Environment. J. Mol. Liq..

[47] Abdallah M., Al Bahir A., Altass H., Fawzy A., El Guesmi N., Al-Gorair A.S., Benhiba F., Warad I., Zarrouk A. (2021). Anticorrosion and adsorption performance of expired antibacterial drugs on Sabic iron corrosion in HCl solution: Chemical, electrochemical and theoretical approach. J. Mol. Liq..

[48] Abdallah M., Al-Gorair A.S., Fawzy A., Hawsawi H., Hameed R.S.A. (2021). Enhancement of adsorption and anticorrosion performance of two polymeric compounds for the corrosion of SABIC carbon steel in hydrochloric acid. J. Adhes. Sci. Technol..

[49] Sayed S.Y., El-Deab M.S., El-Anadouli B.E., Ateya B.G. (2003). Synergistic effects of ben- zotriazole and copper ions on the electrochemical impedance spectroscopy and corrosion behavior of iron in sulfuric acid. J. Phys. Chem. B.

[50] Manjula P., Manonmani S., Jayaram P., Rajendran S. (2001). Corrosion behaviour of carbon steel in the presence of N-cetyl-N,N,N-trimethylammonium bromide, Zn_2_^+^ and calcium gluconate. Anti-Corros. Methods Mater..

[51] Bjelopavlic M., Ralston J., Reynolds G. (1998). Adsorption of Monoalkyl Phosphates at the Zircon–Aqueous Solution Interface. J. Coll. Int. Sci..

[52] Singh A., Ansari K., Haque J., Dohare P., Lgaz H., Salghi R., Quraishi M. (2018). Effect of electron donating functional groups on corrosion inhibition of mild steel in hydrochloric acid: Experimental and quantum chemical study. J. Taiwan Inst. Chem. Eng..

[53] Melendres C.A., Camillone N., Tipton T. (1989). Laser raman spectroelectrochemical studies of anodic corrosion and film formation on iron in phosphate solutions. Electrochim. Acta.

[54] Toghan A., Abo-baker A.M., Rageh H.M., Abd-Elsabour M. (2019). Green Electrochemical Strategy for One-step Synthesis of New Catechol Derivatives. RSC Adv..

[55] Alfakeer M., Abdallah M., Fawzy A. (2020). Corrosion inhibition effect of expired ampicillin and flucloxacillin drugs for mild steel in aqueous acidic medium. Int. J. Electrochem. Sci..

[56] Abdallah M., Fawzy A., Alfakeer M. (2020). Inhibition potentials and adsorption performance of two sulfonylurea antibiotic expired drugs on the corrosion of mild steel in 0.5 M H_2_SO_4_. Int. J. Electrochem. Sci..

[57] Christov M., Popova A. (2004). Adsorption characteristics of corrosion inhibitors from corrosion rate measurements. Corros. Sci..

[58] Fawzy A., Toghan A. (2021). Inhibition Evaluation of Chromotrope Dyes for the Corrosion of Mild Steel in an Acidic Environment: Thermodynamic and Kinetic Aspects. ACS Omega.

[59] Shukla S.K., Quraishi M. (2009). Cefotaxime sodium: A new and efficient corrosion inhibitor for mild steel in hydrochloric acid solution. Corros. Sci..

[60] Abdallah M., Sobhi M., Altass H. (2016). Corrosion inhibition of aluminum in hydrochloric acid by pyrazinamide derivatives. J. Mol. Liq..

[61] Shaban M.M., Negm N., Farag R., Fadda A., Gomaa A.E., Farag A., Migahed M. (2022). Anti-corrosion, antiscalant and anti-microbial performance of some synthesized trimeric cationic imidazolium salts in oilfield applications. J. Mol. Liq..

[62] Zhao T.P., Mu G.N. (1999). The adsorption and corrosion inhibition of anion surfactants on aluminium surface in hydrochloric acid. Corros. Sci..

[63] Bentiss F., Traisnel M., Lagrenee M. (2000). The substituted 1,3,4-oxadiazoles: A new class of corrosion inhibitors of mild steel in acidic media. Corros. Sci..

[64] Durnie W., De Marco R., Jefferson A., Kinsella B. (1999). Development of a Structure-Activity Relationship for Oil Field Corrosion Inhibitors. J. Electrochem. Soc..

[65] ElAchouri M., Hajji M.S., Salem M., Kertit S., Aride J., Coudert R., Essassi E. (1996). Some Nonionic Surfactants as Inhibitors of the Corrosion of Iron in Acid Chloride Solutions. Corrosion.

[66] Xu B., Liu Y., Yin X., Yang W., Chen Y. (2013). Experimental and theoretical study of corrosion inhibition of 3-pyridinecarbozalde thiosemicarbazone for mild steel in hydrochloric acid. Corros. Sci..

[67] Bockris J.O.M., Reddy A.K.N. (1977). Modern Electrochemistry, 2.

[68] Marsh J. (1988). Advanced Organic Chemistry.

[69] Migahed M.A., Farag A.A., Elsaed S.M., Kamal R., Abd El-Bary H. Corrosion inhibition of carbon steel in formation water of oil wells by some schiff base non ionic surfactants. Proceedings of the EUROCORR 2009.

[70] Noor E.A. (2005). The inhibition of mild steel corrosion in phosphoric acid solutions by some N-heterocyclic compounds in the salt form. Corros. Sci..

[71] Aoun S.B. (2013). Highly Efficient Corrosion Inhibition of Carbon Steel in Aggressive Acidic Media with a Pyridazinium-based Ionic Liquid. Int. J. Electrochem. Sci..

[72] Fawzy A., Farghaly T.A., El-Ghamry H.A., Bawazeer T.M. (2019). Investigation of the inhibition efficiencies of novel synthesized cobalt complexes of 1,3,4-thiadiazolethiosemicarbazone derivatives for the acidic corrosion of carbon steel. J. Mol. Struct..

[73] Hashem H.E., Farag A.A., Mohamed E.A., Azmy E.M. (2022). Experimental and theoretical assessment of benzopyran compounds as inhibitors to steel corrosion in aggressive acid solution. J. Mol. Struct..

[74] Mohamed E.A., Hashem H.E., Azmy E.M., Negm N.A., Farag A.A. (2021). Synthesis, structural analysis, and inhibition approach of novel eco-friendly chalcone derivatives on API X65 steel corrosion in acidic media assessment with DFT & MD studies. Environ. Technol. Innov..

[75] Jessima S.H.M., Subhashini S., Berisha A., Oral A., Srikandan S.S. (2021). Corrosion mitigation performance of disodium EDTA functionalized chitosan biomacromolecule—Experimental and theoretical approach. Int. J. Biol. Macromol..

[76] Cao Z., Tang Y., Cang H., Xu J., Lu G., Jing W. (2014). Novel benzimidazole derivatives as corrosion inhibitors of mild steel in the acidic media. Part II: Theoretical studies. Corros. Sci..

[77] Olasunkanmi L.O., Obot I.B., Kabanda M.M., Ebenso E.E. (2015). Some Quinoxalin-6-yl Derivatives as Corrosion Inhibitors for Mild Steel in Hydrochloric Acid: Experimental and Theoretical Studies. J. Phys. Chem. C.

[78] Farag A.A., Eid A.M., Shaban M.M., Mohamed E.A., Raju G. (2021). Integrated modeling, surface, electrochemical, and biocidal investigations of novel benzothiazoles as corrosion inhibitors for shale formation well stimulation. J. Mol. Liq..

[79] Shahraki M., Dehdab M., Elmi S. (2016). Theoretical studies on the corrosion inhibition performance of three amine derivatives on carbon steel: Molecular dynamics simulation and density functional theory approaches. J. Taiwan Inst. Chem. Eng..

[80] Haque J., Srivastava V., Chauhan D.S., Lgaz H., Quraishi M.A. (2018). Microwave-Induced Synthesis of Chitosan Schiff Bases and Their Application as Novel and Green Corrosion Inhibitors: Experimental and Theoretical Approach. ACS Omega.

[81] Farag A.A., Abdallah H.E., Badr E.A., Mohamed E.A., Ali A.I., El-Etre A. (2021). The inhibition performance of morpholinium derivatives on corrosion behavior of carbon steel in the acidized formation water: Theoretical, experimental and biocidal evaluations. J. Mol. Liq..

[82] Verma C., Quraishi M.A., Kluza K., Makowska-Janusik M., Olasunkanmi L.O., Ebenso E.E. (2017). Corrosion inhibition of mild steel in 1M HCl by D-glucose derivatives of dihydropyrido [2,3-d:6,5-d′] dipyrimidine-2, 4, 6, 8(1H,3H, 5H,7H)-tetraone. Sci. Rep..

[83] Kadhim A., Al-Okbi A.K., Jamil D.M., Qussay A., Al-Amiery A.A., Gaaz T.S., Kadhum A.A.H., Mohamad A.B., Nassir M.H. (2017). Experimental and theoretical studies of benzoxazines corrosion inhibitors. Results Phys..

[84] Farag A.A., Ismail A.S., Migahed M. (2015). Inhibition of carbon steel corrosion in acidic solution using some newly polyester derivatives. J. Mol. Liq..

[85] Huong D.Q., Duong T., Nam P.C. (2019). Effect of the Structure and Temperature on Corrosion Inhibition of Thiourea Derivatives in 1.0 M HCl Solution. ACS Omega.

[86] Mulle U. (2006). Inorganic Structure Chemistry.

[87] Trabanelli G., Carassiti V. (1970). Advances in Corrosion Science and Technology.

